# X-ray driven and intrinsic dynamics in protein gels

**DOI:** 10.1038/s41598-023-38059-z

**Published:** 2023-07-08

**Authors:** Sonja Timmermann, Nimmi Das Anthuparambil, Anita Girelli, Nafisa Begam, Marvin Kowalski, Sebastian Retzbach, Maximilian Darius Senft, Mohammad Sayed Akhundzadeh, Hanna-Friederike Poggemann, Marc Moron, Anusha Hiremath, Dennis Gutmüller, Michelle Dargasz, Özgül Öztürk, Michael Paulus, Fabian Westermeier, Michael Sprung, Anastasia Ragulskaya, Fajun Zhang, Frank Schreiber, Christian Gutt

**Affiliations:** 1 Department Physik, Universität Siegen, Walter-Flex-Str. 3, 57072 Siegen, Germany; 2 Deutsches Elektronen-Synchrotron DESY, Notkestr. 85, 22607 Hamburg, Germany; 3 Institut für Angewandte Physik, Universität Tübingen, Auf der Morgenstelle 10, 72076 Tübingen, Germany; 4 Fakultät Physik/DELTA, TU Dortmund, Maria-Goeppert-Mayer-Str. 2, 44227 Dortmund, Germany

**Keywords:** Proteins, Condensed-matter physics, Techniques and instrumentation

## Abstract

We use X-ray photon correlation spectroscopy to investigate how structure and dynamics of egg white protein gels are affected by X-ray dose and dose rate. We find that both, changes in structure and beam-induced dynamics, depend on the viscoelastic properties of the gels with soft gels prepared at low temperatures being more sensitive to beam-induced effects. Soft gels can be fluidized by X-ray doses of a few kGy with a crossover from stress relaxation dynamics (Kohlrausch–Williams–Watts exponents $k \approx 1.5$ to 2) to typical dynamical heterogeneous behavior ($k<$1) while the high temperature egg white gels are radiation-stable up to doses of 15 kGy with $k\ge 1.5$. For all gel samples we observe a crossover from equilibrium dynamics to beam induced motion upon increasing X-ray fluence and determine the resulting fluence threshold values $\Phi _D$. Surprisingly small threshold values of $\Phi _D=(3 \pm 2)\times 10^{-3} \,\textrm{ph}\,$ s$^{-1}$ nm$^{-2}$ can drive the dynamics in the soft gels while for stronger gels this threshold is increased to $\Phi _D=(0.9 \pm 0.3) \,\textrm{ph}$ s$^{-1}$ nm$^{-2}$. We explain our observations with the viscoelastic properties of the materials and can connect the threshold dose for structural beam damage with the dynamic properties of beam-induced motion. Our results suggest that soft viscoelastic materials can display pronounced X-ray driven motion even for low X-ray fluences. This induced motion is not detectable by static scattering as it appears at dose values well below the static damage threshold. We show that intrinsic sample dynamics can be separated from X-ray driven motion by measuring the fluence dependence of the dynamical properties.

## Introduction

Understanding the effects of X-ray radiation on structure and dynamics is important for many scientific fields using synchrotron and X-ray free electron laser (XFEL) sources. Biological and soft matter systems are especially sensitive to radiation damage which is today the limiting factor in the accuracy of structure determination using highly intense synchrotron sources^1–10^. Two important pathways of X-ray–matter interactions are direct damage to the molecules themselves^11^ or indirect damage for example via the radiolysis of water and subsequent chemical reactions with the radiolysis byproducts^12–15^. However, the processes and phenomena involved are not fully understood yet which is partly due to its complex time-dependent chemistry and high sample specificity.

An important parameter for estimating and quantifying radiation effects is the overall amount of absorbed energy per mass, the X-ray dose ${\mathcal {D}}$. In small-angle X-ray scattering (SAXS) experiments at room temperature with macro-molecular proteins dissolved in aqueous environments, X-ray doses on the order of kGy are often enough to induce changes to the protein structure^16^. In macro-molecular X-ray crystallography (MX) cryo-cooling reduces the diffusion of radicals allowing dose values as high as 10–100 MGy before a degradation of Bragg peaks is observed^17–21^. Most other soft matter materials show degradation at X-ray doses between kGy and MGy^22^. These dose values can easily be achieved in less than one second of exposure to the X-ray beam from a 3rd generation synchrotron light source. Thus the question of radiation damage is omnipresent in today’s X-ray scattering experiments.

The role of the *rate* of energy deposition, the dose rate ${\mathscr {D}}$, on diffraction properties is less clear. In MX at synchrotron sources, cryo-cooling seems to prevent dose rate effects^8,23^, while in other cases employing high dose rates may help to run out effects of radiation damage^24,25^. However, high dose rates can also induce X-ray driven dynamics in samples^26–30^ that can also be used as a new probe to explore otherwise inaccessible dynamic modes for example in frozen-in samples^31^. Such accelerated dynamics is especially relevant when investigating dynamic and kinetic phenomena in X-ray scattering experiments, for example near phase transitions^32–36^, as thresholds for beam induced dynamics may be considerably lower than those reported for structural changes. Importantly, for experiments seeking to study equilibrium dynamics it is an additional challenge to separate intrinsic sample dynamics from beam induced effects, in particular as the very nature of beam induced dynamics and its relation to structural changes, sample properties and damage thresholds is not fully clear yet.

Here, we use X-ray photon correlation spectroscopy (XPCS)^33,37–44^ to investigate simultaneously the effects of dose and dose rate on both, structure and dynamics, of egg white protein gel samples on the length scale of the gel’s mesh size (hundreds of nm). We find that the viscoelastic properties of the gel-network seem to play a key role in the structural–dynamical response of the sample to the X-ray radiation. Soft egg white gels prepared at temperatures at and below 70 $^{\circ }$C can be fluidized by the X-ray dose. A higher preparation temperature creates a higher density of crosslinks in the gel^45–47^ such that these strong gels are considerably more stable under the beam. In contrast, soft gels are found to be very susceptible to beam induced dynamics and we observe the crossover from intrinsic sample dynamics to beam induced motions at quite small dose rates of 0.006 kGy s${^{-1}}$ while stronger gels require dose rates of 0.6 kGy s${^{-1}}$ and beyond to drive the dynamics. We determine maximum values of X-ray doses needed for such X-ray induced decorrelations and find them to be close to the structural damage thresholds suggesting a link between driven dynamics and structural damage.

Comparing our results to published data on driven dynamics reveals that the susceptibility for X-ray driven motion in the soft egg white gels is orders of magnitude larger than those found for example in oxide glasses^26^ or protein glasses^30^. As the dynamics of glasses and gels on the respective length scales arise from stress-relaxation events, we hypothesize that the elastic response to an absorbed X-ray photon and thus the elastic moduli play an important role in this process. This could explain the very different susceptibility to X-ray induced motion across the systems investigated. Our findings not only yield new insights into X-ray-matter interactions but also may help to design adapted measurement schemes which is especially important for the high coherent flux of 4th generation light sources^41,48,49^.

## Results

### Measurement scheme and data collection

**Figure 1:**
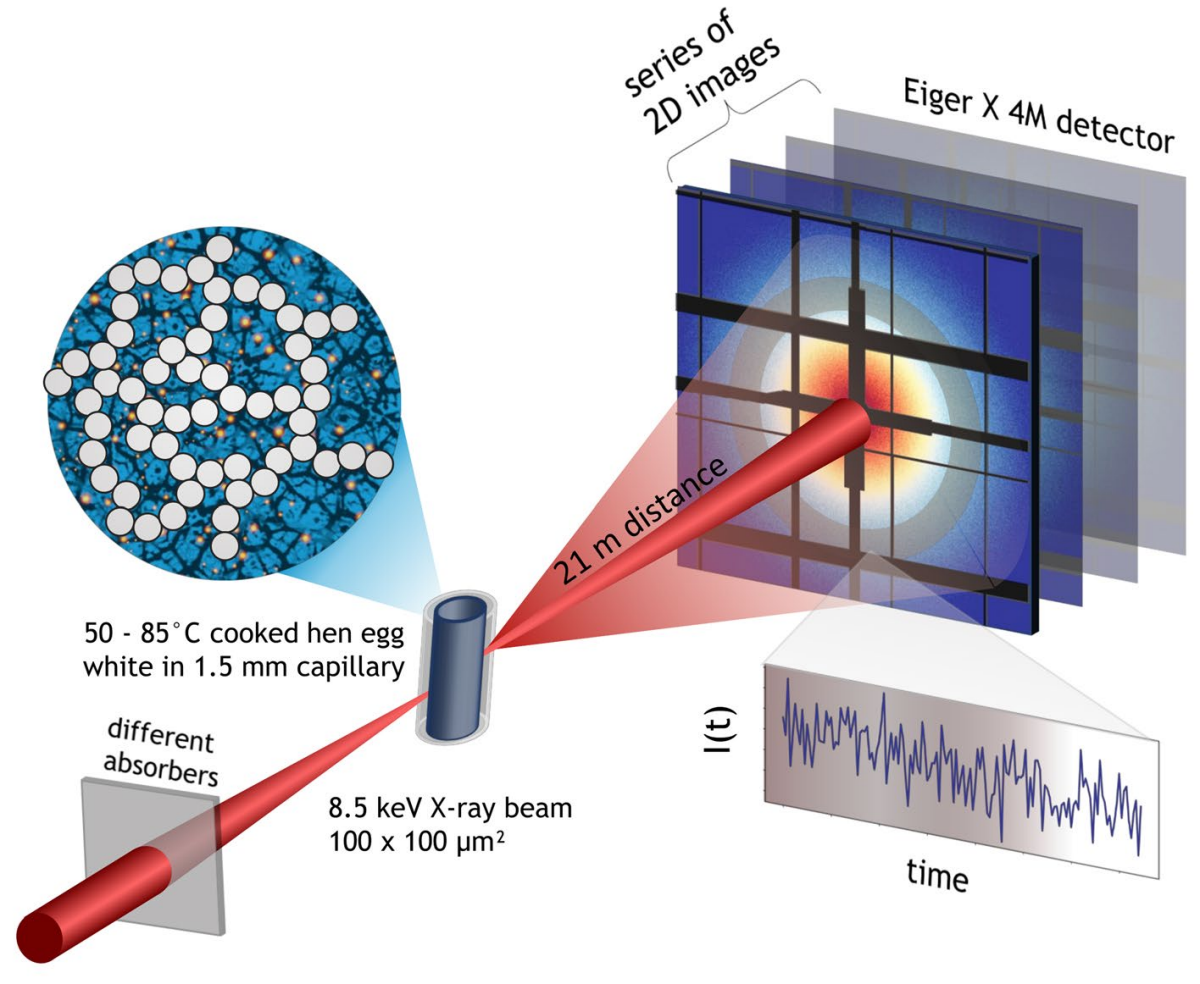
Schematics of the experiment. Hen egg white was filled in 1.5 mm quartz capillaries and heated to create a protein gel. The measurements were performed at beamline P10 at PETRA III (DESY) at 8.5 keV photon energy and a beam size of 100 $\times$ 100 $\upmu {\textrm{m}}^{2}$. We varied the incident coherent photon fluence by inserting different sets of silicon wafers and collected time series of speckle patterns 21.2 m downstream of the sample with an Eiger X 4M detector. Correlating the fluctuating intensities from the speckles gives access to the dynamics of the sample (figure created with^78^).

The experiments were performed at the coherence applications beamline P10 of PETRA III (DESY). The hen egg white was filled into quartz capillaries of diameter 1.5 mm and samples were heated to temperatures in the range 50–85 $^{\circ }$C, which covers the denaturation temperatures of the different protein components in the egg white^45,50^. After heating for 40 min the samples were left at room temperature for about 1 h. The XPCS measurements were performed in the USAXS (ultra-small angle X-ray scattering) setup with a distance of 21.2 m between the sample holder and the Eiger X 4M detector (75 $\times$ 75 $\upmu {\textrm{m}}^{2}$ pixel size) that records time series of the scattered intensity (Fig. 1). A photon energy of 8.54 keV ($\lambda$ = 1.45 Å) was used. The range of wave vector transfers *q* of 0.005–0.2 nm$^{-1}$ gives access to the dynamics on the length scale of the gel’s mesh size^51^. The photon fluence is controlled by inserting absorbers with different thickness before the sample (see “Methods”).

### Static ultra small angle X-ray scattering

**Figure 2:**
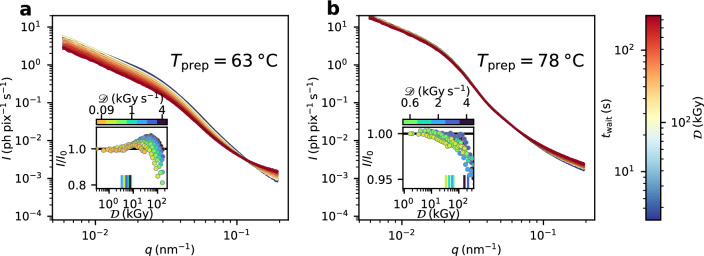
Dose and dose rate effects on the static scattering. (**a**) Scattered intensity of egg white samples prepared at 63 $^{\circ }$C. (**b**) Scattered intensity of egg white samples prepared at 78 $^{\circ }$C. Both samples were irradiated with a fluence of $\Phi = 6$ ph s$^{-1}$ nm$^{-2}$ (dose rate $\approx$ 4 kGy s$^{-1}$) for 200 s such that the sample accumulates a total dose of 800 kGy during the measurement. The color indicates the measurement time and thereby the accumulated dose. Insets: comparison of changes in the scattered intensity for different dose rates indicated by different colors. Each data point corresponds to a single intensity curve like those shown in the panel figure. The intensity is averaged in the *q*-interval 0.006–0.03 nm$^{-1}$ and normalized to the intensity at the beginning of the measurement $I_0$. The data points in dark blue in the background are those matching the dose rate presented in the panel figures. The small vertical lines close to the x-axis indicate the dose values where the deviation of relative intensity exceeds 1%.

We access the structural changes in the egg white gel caused by radiation effects via temporal changes in the scattered intensity. Figure 2 displays the time and dose resolved azimuthally integrated X-ray scattering intensity for egg white samples prepared at 63 $^{\circ }$C (Fig. 2a) and 78 $^{\circ }$C (Fig. 2b). The series depicted in the panel figures were recorded with a dose rate of 4 kGy s${^{-1}}$ reaching a maximum accumulated dose of 800 kGy. We observe significant X-ray-induced changes to the gel structure for both samples with the structure of the low temperature sample (Fig. 2a) being more sensitive to dose than the high temperature sample (Fig. 2b). Intensity curves for other temperatures and dose rates are presented in Fig. S3 in the SI. The effects of the accumulated dose on the structure are shown in the insets of Fig. 2 by plotting relative changes to the intensity ($I(t)/I_0(t=0)$) averaged in the *q*-interval from 0.006 to 0.03nm$^{-1}$. The data points obtained from Fig. 2a,b (in dark blue) can be compared to other measurements performed with smaller dose rates that are shown in different colors as indicated by the colorbar above the inset.

The effects of the dose rate on the structure are shown in the insets of Fig. 2 by plotting relative changes to the intensity ($I(t)/I_0(t=0)$) averaged in the *q*-interval from 0.006 to 0.03 nm$^{-1}$. This interval covers the region of the most apparent changes to *I*(*q*) and is identical to the range of *q*-values available for the XPCS analysis of the dynamics.

The plots reveal that the structure of the low temperature egg white sample ($T_{\textrm{prep}}$ = 63 $^{\circ }$C, Fig. 2a) starts to degrade after a dose of a few kGy visible via a strong increase in the relative intensity. We define the threshold value for radiation induced structural changes via the dose value at which the relative changes in scattering intensity exceed 1%, similar to Reiser et al.^24^. These threshold dose values as indicated by vertical lines in the inset of Fig. 2a are in the range of 3–9 kGy and nearly independent of the applied dose rate. Upon accumulating further dose the scattered intensity reaches a maximum before it starts to decrease. The position and the value of this maximum depend on the applied dose rate with higher dose rates leading to a more pronounced maximum at higher dose values. The other samples prepared at 63–70 $^{\circ }$C show a similar trend (see SI material).

A different behavior is observed for the samples prepared at temperature of 73 $^{\circ }$C and above (Fig. 2b). Here, a decrease in scattered intensity becomes evident only at higher dose values of 15–150 kGy without notable increase in intensity in-between. Moreover, the threshold values of dose induced changes depend on the dose rate. Applying high dose rates results in radiation induced changes appearing at higher dose values which we will discuss later in view of the sample dynamics.

We attribute the observed overall differences in radiation susceptibility of both egg white samples to the different content of denaturated proteins. Hen egg white consists of approximately 40 different proteins^45,50,52,53^ among which ovalbumin (54%)^54–57^ and ovotransferrin (12%)^58,59^ represent the largest fractions. The denaturation temperature of ovotransferrin is in the range of 61–69 $^{\circ }$C^45,50,60^ while ovalbumin typically denaturates at temperatures slightly above 70 $^{\circ }$C^46,61^. In addition to the higher volume fraction of denaturated proteins above 70 $^{\circ }$C, ovalbumin forms a stronger gel network than ovotransferrin due to the exposure of its sulfhydryl groups^45–47^. Subsequently, all egg white samples prepared at temperatures $T_{\textrm{prep}} \ge$73 $^{\circ }$C display a reduced susceptibility to radiation damage (see SI material). In contrast, the higher radiation sensitivity of the 63 $^{\circ }$C egg white sample is representative for all samples in the temperature range 63–70 $^{\circ }$C which form the weakly linked gel network of denaturated ovotransferrin. As a consequence of the findings in this analysis and for reasons of simplicity, we will refer to the samples prepared at 63–70 $^{\circ }$C as soft gel networks and to the samples prepared at 73–85 $^{\circ }$C as strong gel networks.

### X-ray photon correlation spectroscopy

To understand the processes involved we need to resolve also the dynamic processes that accompany the absorption of ionizing radiation. For this we make use of the high coherence of the synchrotron facility PETRA III and perform XPCS experiments to evaluate the influence of X-ray irradiation on the dynamics via two-time correlation functions (TTCs).

Illuminating a disordered egg white sample with a coherent X-ray beam produces a speckle pattern^62–64^ that fluctuates according to the microscopic motion of the protein gel. We correlate these fluctuating intensities *I*(*q*, *t*) at time $t_1$ and $t_2$ in a TTC to obtain information on the dynamics^37,65^:

1 $$\begin{aligned} c^{(2)}(q,t_1,t_2)=\frac{\langle I_p(q,t_1)I_p(q,t_2)\rangle _p}{\langle I_p(q,t_1)\rangle _p\langle I_p(q,t_2)\rangle _p}. \end{aligned}$$

Here, ${\langle \dots \rangle _p}$ represents the average over all detector pixels within an annulus of average momentum transfer *q*.

**Figure 3:**
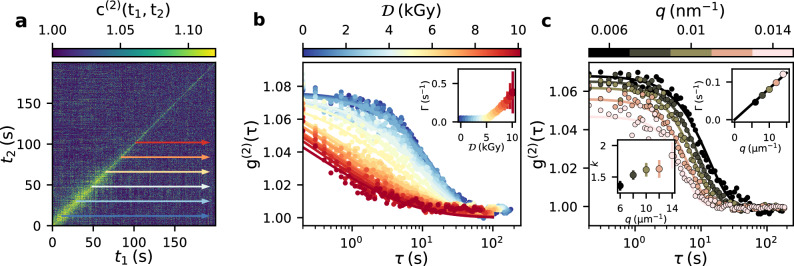
Correlation analysis of dynamics. (**a**) TTC calculated at $q=$ 0.006 nm$^{-1}$. The arrows indicate the temporal position and direction of the cuts for extracting the $\textrm{g}^{(2)}$ functions. The egg white sample was prepared at 63 $^{\circ }$C and measured with an X-ray fluence of $\Phi$ = 0.14 ph s$^{-1}$ nm$^{-2}$ (dose rate $\approx$ 0.09 kGy s$^{-1}$). (**b**) Resulting $\textrm{g}^{(2)}$ functions for different waiting times $t_2$. Solid lines represent fits with a KWW function (Eq. (2)). Inset: relaxation rates $\Gamma$ obtained from the fit. The x-axis displays the dose equivalents of the waiting time $t_2$. (**c**) Comparison of $\textrm{g}^{(2)}$ functions starting at a dose of 1 kGy for different momentum transfers *q*. The solid line in the upper right inset figure represents a linear function $\Gamma \propto q$. The corresponding values of the KWW exponent *k* are displayed in the lower left inset.

Figure 3a shows an example of a TTC obtained from an egg white gel prepared at 63 $^{\circ }$C and measured with a dose rate of 0.09 kGy s${^{-1}}$. The TTC decays with increasing distance from the diagonal ($t_1=t_2$) which represents the increasing decorrelation of the speckle patterns due to the sample dynamics. We also observe a decreasing width of the correlation function with increasing measurement time indicating a radiation induced speed-up of the dynamics caused by the accumulated dose.

To quantify this dose effect we extract $g^{(2)}(q,\tau )$ intensity autocorrelation functions via horizontal cuts starting from the diagonal of the TTC at different times $t_2$ and define $\tau =t_1-t_2$ with $t_1 \ge t_2$. The dose equivalents of these starting times depend on the photon fluence $\Phi$ via ${\mathcal {D}}\propto t_2 \cdot \Phi$ and serve here as dose labels for the $g^{(2)}$ functions, noting that the dose increases further with progressing $t_1$ for each point of the correlation function. The correlation functions are modeled by a Kohlrausch–Williams–Watts (KWW) function^66^:

2 $$\begin{aligned} g^{(2)}(q, \tau ) = 1 +\beta (q)\,e^{-2(\Gamma (q)\tau )^k}, \end{aligned}$$

where $\beta (q)$ is the *q*-dependent speckle contrast^67^ ($\beta (q={0.02}\,\text {nm}^{-1}) \approx {9.7}\%$), $\Gamma (q)$ is the decay rate and *k* is the KWW exponent containing information about the type of motion^33,43^.

Figure 3b shows $g^{(2)}$ functions and corresponding KWW fits from the TTC in Fig. 3a. We observe that for the 63 $^{\circ }$C sample the resulting decay rates $\Gamma$ (Fig. 3b inset) increase rapidly after an accumulation of a few kGy (inset of Fig. 3b). Figure 3c displays the *q*-dependence of the $g^{(2)}$ functions at an identical starting dose of 1 kGy. The corresponding decay rates (Fig. 3c, inset) reveal a linear dependence $\Gamma = v\cdot q$ with *v* denoting a velocity. Together with the observed values of the KWW exponents $\ge 1.5$ (lower left inset in Fig. 3c) this type of ballistic motion is typical for gels and connected with stress relaxation after gel formation^51,68–72^. We will later use this fluence- and sample-dependent velocity to compare fluence effects independent of the wave vector transfer *q*.

### Dose effects on dynamics

**Figure 4:**
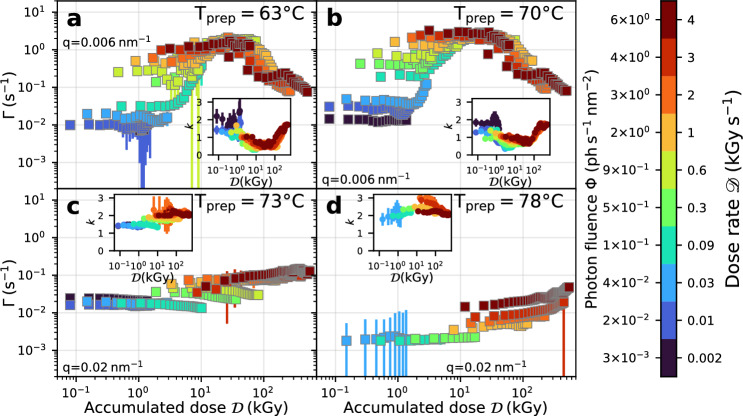
Radiation effects on relaxation rates. Dose and dose rate effects on the relaxation rates $\Gamma$ of egg white gel samples prepared at temperatures 63 $^{\circ }$C (**a**), 70 $^{\circ }$C (**b**), 73 $^{\circ }$C (**c**) and 78 $^{\circ }$C (**d**). All measurements have been performed with the samples cooled down to room temperature. The decay rates $\Gamma$ are shown as a function of dose ${\mathcal {D}}$ for ten different photon fluences/dose rates. The data for 63 $^{\circ }$C and 70 $^{\circ }$C were analyzed at $q = {0.006}$ nm$^{-1}$. For the analysis of the samples prepared above 70 $^{\circ }$C, *q* was increased to 0.02 nm$^{-1}$ due to the slower decays of the $g^{(2)}$ functions. The insets show the KWW parameter *k* as a function of dose and dose rate. The color indicates the X-ray fluences and dose rates.

We apply this XPCS analysis procedure to all samples in the temperature range 63–85 $^{\circ }$C that we measured with ten different dose rates in the range from 0.002 to 4 kGy s${^{-1}}$, which are the same measurements as in the analysis of the static scattering above. The resulting values of the decay rates $\Gamma$ as a function of dose and dose rate are displayed in Fig. 4 for four different preparation temperatures, noting that all measurements have been performed at room temperature. We observe a similar behavior in the dynamics as seen in the structural changes with the soft gel network samples (Fig. 4a,b) being more susceptible to dose effects than the ones forming a strong gel network (Fig. 4c,d).

In the temperature range 63–70$^{\circ }$C the decay rates are almost constant until a dose of few kGy. Crossing this threshold dose value, the relaxation rate strongly increases by almost two orders of magnitude implying a fluidization of the soft gels formed by the heat denaturated ovotransferrin. This fluidization is accompanied by a decrease of KWW exponents to values $k<1$ (insets in Fig. 4). The reverse is usually observed during the aging of a gel, i.e. rates decrease and KWW exponents increase until stress relaxation is the final mechanism of dynamics^73^. At the same time we observe a transition from ballistic ($\Gamma \propto q$) to diffusive motion ($\Gamma \propto q^2$). Thus, the data suggests that the gel structure is fluidized under the influence of the radiation and becomes mobile again. This also explains the increase in the static scattering signal (inset of Fig. 2a) at doses ${\mathcal {D}}$ of 10–50 kGy as the fluidized gel is capable of further aggregation.

Beyond a dose of 10 kGy the decay rate reaches a dose rate-independent maximum value. This maximum is followed by a slow-down of the dynamics of one order of magnitude which starts around $\approx {50}$ kGy to the highest recorded doses of $\approx {600}$ kGy. This decrease sets in at lower doses for higher dose rates. The slow down is accompanied by an increase of the KWW exponents back to values of $k \approx 1.5$ indicating that stress relaxation is re-established as the main relaxation mechanism for very high doses. This points towards a radiation induced denaturation of the remaining protein content, mostly ovalbumin, with the subsequent formation of a gel.

A different picture emerges for the samples with a strong network (Fig. 4c,d) that have been prepared at temperatures of 73 $^{\circ }$C and above. Here, the influence of the accumulated dose on the dynamics is much less pronounced. A maximum speed-up of the decay rate $\Gamma$ by a factor of five becomes visible for $T_{\textrm{prep}}$ = 73 $^{\circ }$C (Fig. 4c) for the highest dose rates of 4 kGy s${^{-1}}$ and doses between 10 and 100 kGy. The KWW exponents vary only slightly (insets of Fig. 4c,d) indicating that the dynamics are always stress driven relaxation.

Generally speaking, our results demonstrate that the strong protein gels formed at higher temperatures are also dynamically much more stable under irradiation. Soft protein gels show dose induced fluidization for low doses followed by a slow-down due to radiation induced denaturation with further gel formation.

### Dose rate effects and beam-induced dynamics

**Figure 5:**
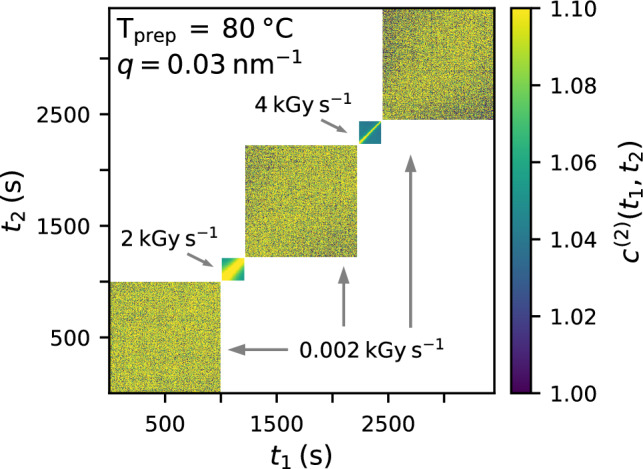
Dose rate effect on dynamics. Two-time correlation function (TTC) from a continuous illumination of a single spot on an egg white sample prepared at 80 $^{\circ }$C with changing dose rates during the exposure. The corresponding values of the dose rates are indicated in the figure.

We investigate the nature of dose rate effects on the dynamics by continuously illuminating a single spot on an egg white sample while changing absorbers during the scan thus changing the dose rates. Examples of resulting TTCs are shown in Fig. 5 where scans with high dose rates of 4 kGy s${^{-1}}$ and 2 kGy s${^{-1}}$ are enclosed by scans with a smaller dose rate of 0.002 kGy s${^{-1}}$. It can be seen that the almost frozen-in slow dynamics of the 80 $^{\circ }$C-sample measured at the low dose rate is instantly accelerated when applying the higher dose rate but returns also instantly back to the slow dynamics as soon as the dose rate is decreased again. On top of this dose rate induced switching of the dynamics we observe a slower dose induced speed-up of the overall dynamics when comparing the first and the last TTC in Fig. 5 in agreement with the results from Fig. 4d.

Such flux dependent dynamics have been observed before in XPCS experiments on oxide and network glasses^26–29^, albeit with orders of magnitude higher dose rates, and also recently in dense protein glasses^30^. In our case the dynamics of the protein gels are governed by stress relaxations in which the correlation functions can be modeled as a series of consecutive relaxation events in the stressed material^68,74^. These relaxation events occur with a rate $\gamma$ and an average displacement step of size $\delta$. With this the $g^{(2)}$ function can be modeled as a sum over displacement events $g^{(2)}(q,t)=\sum _N P(N,t) h(q,N)$ where the Poisson distribution $P(N,t)=\exp (-\gamma t)(\gamma t)^N/N!$ gives the probability of *N* relaxation events occurring during a time interval *t*. A Gaussian distribution $h(q,N)=\exp (-(qN\delta )^2)$ for the decorrelation after *N* events, with typical displacement $\delta$ after a single relaxation event, yields the right values for the KWW parameter and the ballistic type of motion $\Gamma \propto q$ observed in our experiment. From the measured KWW exponents we infer values of $q \delta \approx$ 0.01 to 0.1 (see SI). At these conditions the resulting relaxation rate of the correlation function is connected to the microscopic stress relaxation events via $\Gamma \approx \gamma q \delta$ (see also SI), implying that the typical decorrelation rate $\Gamma$ of the $g^{(2)}$ functions is a factor of 10–100 times smaller than the microscopic stress relaxation rate $\gamma$.

**Figure 6:**
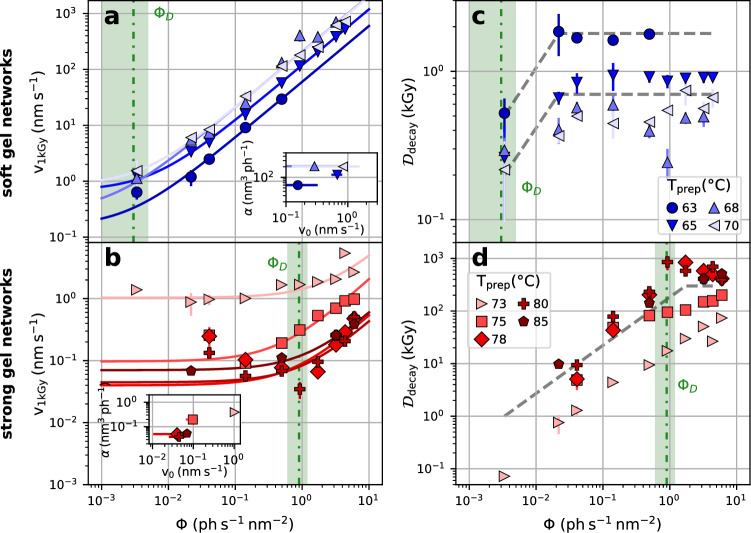
Effect on sample velocity. Comparison of fluence (dose rate) effects on the dynamics of egg white gel samples at a constant starting dose of 1 kGy (see Fig. 4). (**a,c**) Soft gel networks prepared via heating to temperatures T_prep_ = 63–70 $^{\circ }$C. (**b,d**) Strong gel networks prepared by heating to T_prep_ = 73–80 $^{\circ }$C. (**a,b**) Sample velocity $v=\Gamma /q$ as a function of photon fluence $\Phi$. Solid lines represent a fit with $v=v_0+\alpha \Phi$, where $v_0$ represents the equilibrium dynamics. In the inset figures, the fit parameters $v_0$ and $\alpha$ are shown (for details see SI). Vertical green dashed lines are guides to the eye indicating the onset of dynamics induced by the X-ray beam (threshold fluences $\Phi _D$). (**c,d**) Accumulated dose values at which the $g^{(2)}$ functions have decayed to a value of $g^{(2)}(t')=1+\beta \exp (-2)$ assuming $q={0.02}$ nm$^{-1}$ . Gray dashed lines are guides to the eye.

To disentangle dose and dose rate effects we evaluate the dynamics as a function of photon fluence for a fixed starting dose value of 1 kGy. Furthermore, we eliminate the *q*-dependence of the relaxation rates by making use of the ballistic type of motion with $\Gamma =v\cdot q$ which allows to extract the velocity *v* as a *q*-independent indicator of the sample dynamics (see also Fig. 3c). The increase of sample velocity as a function of the incident X-ray fluence $\Phi$ is shown in Fig. 6a for the soft gel networks and in Fig. 6b for the strong gel networks, respectively.

We describe our data with a simple phenomenological model (see e.g.^30^)

3 $$\begin{aligned} v(\Phi ) = v_0 + \alpha \Phi , \end{aligned}$$

in which $v_0$ represents the equilibrium sample velocity, $\Phi$ is the X-ray fluence and $\alpha$ is a material constant describing the strength of the X-ray–matter interaction which accelerates the sample dynamics. This simple model describes the data reasonably well and the resulting fit parameters $\alpha$ and $v_0$ are shown in the insets of Fig. 6 (see SI for details of fit procedures). This linear dependence of the dynamics on photon fluence has been observed in other systems as well^26,28–30^. It implies that the photon fluence induces an additional stress relaxation mechanism with the rate of microscopic events $\gamma$ being proportional to the applied fluence via $\gamma = \alpha \cdot \Phi / \delta$.

We define threshold values for the X-ray fluence $\Phi _D$ via the point where the induced dynamics, $\alpha \Phi _D$ outperform the intrinsic dynamics $v_0$ implying a beam-induced speed up of the dynamics by ${100}\%$. Averaging over all networks of a each type, we find fluence thresholds of $(0.003\pm 0.002)$ ph s$^{-1}$ nm$^{-2}$ for the soft gel networks and $(0.9\pm 0.3)$ ph s$^{-1}$ nm$^{-2}$ for the strong gels (indicated by vertical green sections in Fig. 6). The gel network prepared at ${73}\,^{\circ }$C is excluded from this average due to the larger value of $v_0$ indicating that the network is in an intermediate state between strong to soft gel network.

## Discussion

It is instructive to convert the velocities from Fig. 6 back into decay times of the correlation functions and to calculate the dose equivalents of these decay times. By this approach we consider that the sample accumulates further dose during the measurement of the correlation functions (increasing $t_1$). We choose the momentum transfers in line with the ones used in the analysis of the dose effects, that are $q={0.006}$ nm$^{-1}$ for soft gel networks and $q={0.02}$ nm$^{-1}$ for strong gel networks. We obtain the respective decay times via $t_1'=1/(qv)$ using the velocities at a starting dose of 1 kGy from Fig. 6a,b. Multiplying these decay times $t_1'$ by the different dose rates ${\mathscr {D}}$ (see “Methods” section) yields the corresponding dose values $D_{\textrm{decay}}$ at which the correlation functions decay to a value of $g^{(2)}(t_1')=1+\beta \exp (-2)$ (Fig. 6c,d).

We observe that $D_{\textrm{decay}}$ approaches constant values at fluences $\Phi >\Phi _D$ , indicated by the green lines. Above these fluences the $\textrm{g}^{(2)}$ function is entirely decorrelated by beam induced motion. Adding to ${\mathcal {D}}_{\textrm{decay}}$ the dose value of 1 kGy already received at the start of the correlation yields threshold dose values beyond which all motion is beam-induced.

For the soft gel networks, these maximum doses (i.e. the value where $D_\text{decay}$ levels off with increasing fluence in Fig. 6c,d are between 1.7 and 3 kGy, slightly below the thresholds values found from the analysis of the structural changes. In contrast, the strong gels can be fully decorrelated only at high X-ray fluences with dose values reaching 70–300 kGy. Interestingly, the structural analysis of the strong gels revealed thresholds for structural changes as low as 20 kGy (at dose rates of 0.3 kGy s${^{-1}}$) implying that in the fluence regime of fully beam induced motion the structural changes in the strong gels can occur already during the decorrelation of the correlation function.

In the next step, we estimate radical formation rates from the fluence thresholds $\Phi _D$ that we deduced from the fitting parameters $\alpha$ and $v_0$. We assume that the absorption properties of the protein gels are comparable to the ones of water. In aqueous solutions, the radiolysis products are the main driver for beam damage and an absorption of a 100 eV photon energy results typically in 3 OH radicals^13^. In the irradiated sample volume of a soft gel network, $\Phi _D=(0.003\pm 0.002)\,\textrm{ph}$ s$^{-1}$ nm$^{-2}$ causes a radical formation rate of $\approx 4\times 10^{-7}\,\textrm{radicals}$ s$^{-1}$ nm$^{-2}$ or one radical per second per cube with an edge length of 138 nm. For a strong gel with $\Phi _D=(0.9\pm 0.3)\,\textrm{ph}$ s$^{-1}$ nm$^{-2}$, the same procedure yields a radical formation rate of $\approx 1.1\times 10^{-4}\,\textrm{radicals}$ s$^{-1}$ nm$^{-3}$ or one radical per second per cube with an edge length of 20 nm. The estimates for the radical formation rate in the cuboid example volume yield surprisingly small density rates of radicals required to drive the dynamics instantaneously as it is observed in Fig. 5. This can best be understood considering the open gel network structure of the gels and the nature of the stress driven relaxation which requires to induce only a local breakage of a bond leading then to a coherent elastic response of the network^75^. Thus the few radicals do not move the whole corresponding volume, but rather trigger elastic relaxation events. The length scales found here agree with the reported values of spatial extension of decorrelation events in egg white^51^.

**Figure 7:**
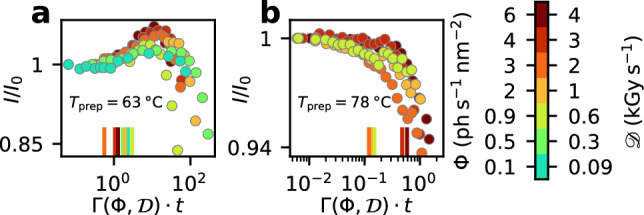
Interplay of structure and dynamics. Evolution of the relative changes in the scattered intensity as a function of the product $\Gamma (\Phi ,{\mathcal {D}}) \cdot t$ of measurement time *t* and decay rate $\Gamma$. The intensity is averaged in the q range from 0.006 to 0.03 nm$^{-1}$ and normalized to the intensity at the beginning of the measurement $I_0$. The fluence-dependent and dose-dependent decay rates have been taken from Fig. 4. (**a**) Soft gel network prepared at T_prep_ = 63 $^{\circ }$C, (**b**) strong gel network prepared at T_prep_ = 78 $^{\circ }$C. The color indicates the X-ray fluence and dose rates; the vertical lines mark the value where the intensity deviation exceeds the 1%-threshold.

More quantitative insights are obtained when plotting the relative changes $I/I_0$ of the scattered intensities not as a function of dose, but as a function of exposure time *t* multiplied by the dose and dose-rate dependent relaxation rate $\Gamma ({\mathcal {D}}, \Phi )$ (Fig. 7). The factor $t \cdot \Gamma ({\mathcal {D}}, \Phi )$ represents via $g^{(2)}(t)=1+\beta \exp (-t \cdot \Gamma ({\mathcal {D}}, \Phi ))$ the degree of correlation during the exposure time *t*. We observe that the relative intensity deviations are falling almost onto a single master curve (see also SI material) indicating that the damage thresholds of structural changes and the respective sample dynamics are connected. We observe that the soft gel networks all display a similar value of $t \cdot \Gamma ({\mathcal {D}}, \Phi ) \approx 1$ at a 1% change to their relative scattering intensity, while for the strong gels this value is smaller with $t \cdot \Gamma ({\mathcal {D}}, \Phi ) \approx 0.1$. With $\Gamma \approx q \delta \gamma$ and values of $q \delta \approx 0.01$ to 0.1 we conclude that for the soft gels changes to the structure appear after $t \cdot \gamma$ = 10–100 stress events while for strong gels this is reduced to 1–10 events which is due to the very high doses applied during the very slow relaxation.

The maximum tolerable dose ${\mathcal {D}}_{\textrm{max}}$, the dose rate ${\mathscr {D}}$ and the dynamics are thus related via ${\mathcal {D}}_{\textrm{max}} \propto {\mathscr {D}} / \Gamma ({\mathcal {D}}, \Phi )$ and it is instructive to examine this relationship for two limiting cases. In the first case, we assume that the relaxation rate $\Gamma$ is independent of dose and dose rate ($\Gamma =\Gamma _0$) which yields a limit ${\mathcal {D}}_{\textrm{max}} \propto {\mathscr {D}} / \Gamma _0$ favoring high dose rates and slow dynamics for achieving high damage thresholds. This is the regime in which, for example, a high X-ray fluence allows to run out radiation damage^24^. In the other limit we assume that the dynamics is beam induced motion only i.e. $\Gamma =\alpha \cdot q \cdot \Phi$ leading to ${\mathcal {D}}_{\textrm{max}} \propto 1 / \alpha q$ which is then independent of dose rate. For the egg white gels $\Gamma$ depends on both dose and dose rate and thus ${\mathcal {D}}_{\textrm{max}}$ as well which explains the data shown in the inset of Fig. 2.

**Figure 8:**
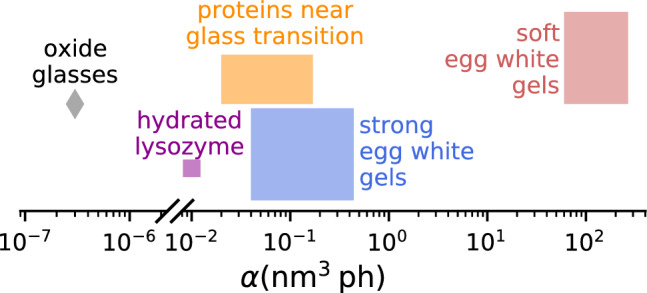
Comparison to other sample systems. Parameter $\alpha$ describing the X-ray-induced acceleration of the dynamics for different sample systems (see Eq. (3)). The plot also includes data from Chushkin et al.^30^ on protein glasses (yellow), Ruta et al.^26^ on oxide glasses (grey) and Bin et al.^79^ who investigated hydrated lysozyme (pink, see SI material for details).

Finally, we can compare our results of $\alpha$ to published work on protein glasses^30^ from which we extract values of $\alpha \approx 2 \times 10^{-2}\,\mathrm {nm^3\,ph^{-1}}$ and to oxide glasses^26^ which gives $\alpha \approx 5 \times 10^{-6}\,\mathrm {nm^3\,ph^{-1}}$ (see Fig. 8). These values are four respectively eight orders of magnitude smaller than the corresponding $\alpha$ for hen-egg white at $\mathrm {T_{\textrm{prep}}}$ = 63$^{\circ }$C. We attribute these large differences and the temperature dependent behavior of $\alpha$ observed in our study to the large differences in viscoelastic properties of the respective materials. Indeed, the relationship $\alpha \propto \gamma \cdot \delta / \Phi$ suggests that materials displaying slow dynamics and small values of elastic displacements $\delta$ will show accordingly small values of $\alpha$. This in turn explains the generally enhanced values of $\alpha$ for softer materials. Rheology measurements of Bonilla and Clausen^76^ revealed a sudden increase in yield stress of cooked egg white when the temperature exceeds 72 $^{\circ }$C. This observation is in good agreement with our observation of soft and strong gels in terms of radiation susceptibility. Clearly, more systematic data on other sample systems and additional work by theory and simulation is needed to fully understand the relationship between sample properties and susceptibility to X-rays, in terms of both induced motion and beam damage.

In summary, our experimental results reveal a rich and complex dynamic response of protein gels to X-ray radiation. The combination of static and dynamic measurements shows that X-ray dose can lead to fluidized faster gels but also to more strongly bonded gels with slower dynamics. We find threshold values of X-ray fluence beyond which the dynamics of the protein gels are driven by X-ray induced stress relaxation. We infer that the susceptibility for X-ray driven motion depends on the viscoelastic properties of the sample. These results are important for experiments using synchrotron radiation and aiming to study kinetic and dynamic phenomena in viscoelastic materials which requires to match experimental time scales to sample time scales. Our findings demonstrate that the X-ray fluence is a key parameter for switching between dynamics driven by the X-ray beam and equilibrium dynamics of the sample.

Importantly, we identify values of dose rates and dose values at which the XPCS signal is reflecting true equilibrium dynamics for protein gels. Our study demonstrates that experimental setups and synchrotron instrumentation which want to make use of the high brilliance of 4th generation light sources need to be able reduce the photon density as effectively as possible. This requires making use of the enlarged coherence lengths of the new sources and perform experiments with large beams. The development of fast X-ray detectors with small pixel sizes and the use of increased sample-detector distances are needed to resolve the small speckles from large X-ray beams.

## Methods

### Sample preparation

We purchased hen eggs at a local supermarket, separated the liquid component of the egg white and filled it in quartz capillaries of diameter 1.5 mm sealed with parafilm. The capillaries were placed in a temperature controlled water bath under an angle of 30$^{\circ }$–40$^{\circ }$ with respect to the water level such that bubbles forming during heating were capable to move to the top part of the capillary. Samples were heated for 40 min at the set temperatures (50–85 $^{\circ }$C) and after this cooled down to room temperature (see also SI material).

### Measurement protocol

The measurements were taken on spots separated by 200 $\upmu$m. For the highest dose rates two measurements were performed: one with a short exposure time (5 ms) to capture fast dynamics and one with a longer exposure time (40 ms) to capture the slow dynamics.

### Calculation of flux, dose rate and dose

The photon flux ${\mathcal {F}}_0=6 \times 10 ^{10}$ ph s$^{-1}$ was distributed over the beam area on the sample of 100 $\upmu$m $\times$ 100 $\upmu$m resulting in a maximum photon fluence of $\Phi _0 = 6$ ph s$^{-1}$ nm$^{-2}$. The flux was further reduced by sets of silicon absorbers with absorber *n* being equal to the thickness of a $n \times 25$ $\upmu$m silicon wafer. The experimental absorption unit is realized as combinations of absorbers of different thicknesses allowing only for certain values of *n* (see Table 1). The reduced flux for absorber *n* is calculated from the X-ray transmission of a single 25 $\upmu$m wafer (73%) via:

4 $$\begin{aligned} {\mathcal {F}}_{\mathrm {red.}}^n = {\mathcal {F}}_0 \cdot (0.73)^n. \end{aligned}$$

The photon fluence on the sample reduces in the same way.

The values of dose and dose rates are obtained from the corresponding water equivalents of the irradiated sample volume $V={100}\,\upmu \text {m}\times {100}\, \upmu \text {m}\times 1.5\,{\textrm{mm}}$. Flux-reducing effects of the capillary walls are not taken into account. The accumulated dose of the sample is calculated using^77^:

5 $$\begin{aligned} {\mathcal {D}}=\frac{t_{\textrm{exp}}{\mathcal {F}}_{\mathrm {red.}}E(1-T)}{V\rho }, \end{aligned}$$

where $t_{\textrm{exp}}$ is the exposure time and ${\mathcal {F}}_{\textrm{red}}$ is the flux calculated from Eq. (4). *E* represents the energy of a single X-ray photon, in this case 8.54 keV, and *T* is the transmission of the sample with sample volume *V*. $\rho$ is the mass density of a water equivalent (1000 kg m$^{-3}$). The transmission *T* of 1.5 mm water for 8.54 keV X-rays is 27.8 %.

The dose rate ${\mathscr {D}}$ is derived from the accumulated dose via division by the exposure time $t_{\textrm{exp}}$ and is given in units of kGy s$^{-1}$:

6 $$\begin{aligned} {\mathscr {D}}=\frac{{\mathcal {F}}_{\textrm{red}}E(1-T)}{V\rho } = \frac{{\mathcal {D}}}{t_{\textrm{exp}}}. \end{aligned}$$

The fluences for the absorber configurations used and the resulting dose rates are given in Table 1.

**Table 1:** Reduced fluxes and fluences for absorber configurations and resulting dose rate.

*n*	${\mathcal {F}}_{\mathrm {red.}}$ (ph s$^{-1}$)	$\Phi _{\mathrm {red.}}$(ph s$^{-1}$ nm$^{-2}$)	${\mathscr {D}}$ (kGy s$^{-1}$)
0	$6\times 10^{10}$	6	4
1	$4.4\times 10^{10}$	4.4	2.9
2	$3.2\times 10^{10}$	3.2	2.1
4	$1.7\times 10^{10}$	1.7	1.1
6	$9\times 10^{9}$	0.9	0.6
8	$5\times 10^{9}$	0.5	0.3
12	$1.4\times 10^{9}$	0.14	0.09
16	$4\times 10^{8}$	0.04	0.03
18	$2\times 10^{8}$	0.02	0.01
24	$3\times 10^{7}$	0.003	0.002

*n* number of 25 $\upmu$m Si wafers inserted to attenuate the beam, ${\mathcal {F}}_{{red.}}$ incident flux, $\Phi _{{red.}}$ fluence on sample, ${\mathscr {D}}$ dose rate. In the text, fluence and dose rates are rounded to one significant digit.

## Supplementary Information

Supplementary Information.

## Data Availability

The processed data are published under https://github.com/STimmermann/XPCS_RadiationEffects.

## Code availability

Analysis scripts are published under https://github.com/STimmermann/XPCS_RadiationEffects.
