# Supplementary material for: X-ray driven and intrinsic dynamics in protein gels
Source: Sci Rep. 2023 Jul 8;13:11048. doi: 10.1038/s41598-023-38059-z (PMC10329714; doi:10.1038/s41598-023-38059-z)
Supplement: Supplementary file 1 — Supplementary Information. [file 41598_2023_38059_MOESM1_ESM.pdf]

Supporting Information: X-ray driven and intrinsic and dynamics in protein gels

Sonja Timmermann et al.

Sample preparation

We purchased a hen egg at a local supermarket and separated the liquid component of the egg white using a large pipette. The filling of the quartz capillaries with a diameter of $1.5\,\mathrm{mm}$ was done with a $0.8\,\mathrm{mm}$ syringe needle. The capillaries were sealed with Parafilm and the uncooked samples were stored in the fridge at $5\,^{\circ}\mathrm{C}$. The samples were cooked in sequence and were taken out of the fridge one hour before cooking. Bubbles in the uncooked sample were removed in advance by manual shaking. The cooking was done in a temperature controlled water bath where the capillaries were hold by tweezers under an angle of 30 to 40° with respect to the surface of the water (Fig. S1a) such that the top part of the capillary was in air. With that bubbles forming during the cooking procedure could move to the top part of the capillary and no water entered into the sample. In earlier experiments we noted a slight gradient of the sample dynamics depending on the position in the capillary when the samples were cooked vertically.

The samples were cooked for $40\,\mathrm{min}$ at the set temperature in the range 50 to $85\,^{\circ}\mathrm{C}$. The water was stirred with a magnetic stirrer at 200rpm (Fig. S1b). The temperature of the water bath was cross-checked with an external thermometer that gave a deviation of $\approx 1\,^{\circ}\mathrm{C}$ towards lower temperatures. In the main text we use the temperature values of the temperature control.

After the cooking, the samples were left at room temperature for $\approx 1\,\mathrm{h}$ to reduce aging effects during the measurement. A subset of the cooked samples is shown in Fig. S1c. While the samples cooked below $60\,^{\circ}\mathrm{C}$ are almost clear, there is a rapid increase in turbidity between $57\,^{\circ}\mathrm{C}$ and $63\,^{\circ}\mathrm{C}$ due to the onset of the denaturation of the ovotransferrin component.

All measurements were performed in the region close to the tip of the capillary.

Supporting information on analysis of static scattering

Additional Information Fig. 2

Figure 2 in the main text shows dose and dose rate effects on the static scattering signal of egg white samples prepared at $63\,^{\circ}\mathrm{C}$ (a) and $78\,^{\circ}\mathrm{C}$ (b). The two preparation temperatures are representative for the temperature ranges 63 to $70\,^{\circ}\mathrm{C}$ (soft gel network from ovotransferrin) and 73 to $85\,^{\circ}\mathrm{C}$ (strong gel network due to denaturated ovalbumin). The dose dependent USAXS curves of the other samples are shown in Fig. S4; the inset plots are shown in Fig. S5. In Fig. 2 in the main text, both samples were irradiated with the highest fluence used in the experiment ($\Phi = 6$ ph $\mathrm{s}^{-1}\,\mathrm{nm}^{-2}$ $\mathscr{D} \approx 4$ $\mathrm{kGy\,s}^{-1}$) for 200 s such that the samples accumulate a total dose of $800\,\mathrm{kGy}$ during the measurement. The color bar uses a logarithmic scale to visualize the increase of intensity at low q in the $63\,^{\circ}\mathrm{C}$ sample.

In the insets we compare changes in the scattered intensity for different dose rates indicated by different colors. The average intensity values are obtained from averaging the azimuthally integrated scattering intensities in the q-interval 0.006 to $0.03\,\mathrm{nm}^{-1}$, that is the interval where we perform the XPCS analyses. In the plot, the intensities are normalized to the intensity at the beginning of the measurement I_0 and the transmission of each absorber setting. We present only the curves for dose rates $\geq 0.09\mathrm{kGy\,s}^{-1}$ as the measurements with smaller dose rates do not reach the dose values necessary for seeing an increase/decrease in intensity. We averaged the data points of each dose rate on a logarithmic scale into 30 data points. The small vertical lines indicate the dose values where the deviation of relative intensity exceeds $1\,\%$.

Additional Information on Fig. 7

Figure 7 in the main text displays the evolution of the relative changes in the scattered intensity as a function of the product $\Gamma(\Phi, \mathcal{D}) \cdot t$ of measurement time t and decay rate Γ. The intensity is averaged in the q range 0.006 to $0.03\,\mathrm{nm}^{-1}$ similar to the insets of Fig. 2 in the main text and normalized to the intensity at the beginning of the measurement I_0 and the transmission of the absorber setup. The equivalents of this figure for the other temperatures are displayed in Fig. S6.

Equilibrium USAXS Profiles for All Temperatures

For every egg white sample we recorded a measurement with a low dose rate of $\mathscr{D} = 10\,\mathrm{Gy\,s}^{-1}$ to compare the ultra-small angle X-ray (USAXS) profiles of all samples at a low dose and dose rate. Figure S2 displays the azimuthally integrated average of the scattered intensity collected in the first 100 frames (0.5 to $20\,\mathrm{s}$ depending on exposure time) of every sample. The accumu-

lated dose is below $0.2\,\mathrm{kGy}$. Upon increasing cooking temperature, the intensity in the low q regime increases by up to three orders of magnitude. This increase matches very well with the turbidity of the egg white samples in Fig. S1c and indicates the temperature induced formation of a protein network via denaturation of the different proteins.

Signal-to-Noise Ratio and Measurement Strategy

The feasibility of an X-ray photon correlation spectroscopy experiment can be determined via the signal-to-noise ratio (SNR) that is given by [1–3]:

$$R_{\mathrm{SN}} = \beta \times I_{\mathrm{pix}} \times \sqrt{N_{\mathrm{pix}} \times N_{\mathrm{fr}} \times N_{\mathrm{rep}}}. \tag{1}$$

Here β is the speckle contrast, that is $\approx 10\,\%$ in our setup. I_{pix} is the average intensity per pixel and N_{pix} is the number of pixels in a certain q-annulus. In our analysis setup, $N_{\mathrm{pix}} \approx 400$ at $q = 0.006\,\mathrm{nm}^{-1}$ and increases up to ≈ 70000 at $q = 0.03\,\mathrm{nm}^{-1}$. N_{fr} is the number of recorded frames in the series and N_{rep} is the number of repetitions. Due to the large beam of $100\,\mathrm{\mu m} \times 100\,\mathrm{\mu m}$ the number of measurement spots on the capillary is limited and we usually perform a single measurement per temperature and fluence.

For the high fluences $\Phi \geq 5 \times 10^{-1}$ ph $\mathrm{s}^{-1}\,\mathrm{nm}^{-2}$, the increase in I_{pix} allows to go for smaller exposure times of $t_{\mathrm{exp}} = 0.005\,\mathrm{s}$ with a sufficient SNR to resolve the dynamics at small doses. We perform a second measurement with the same flux on a fresh spot but with a longer exposure time $t_{\mathrm{exp}} = 0.04\,\mathrm{s}$ to catch also the effects at high doses without difficulties in computing and data storage due to large number of frames in a time series. For the lower fluences $\Phi \leq 1 \times 10^{-1}$ ph $\mathrm{s}^{-1}\,\mathrm{nm}^{-2}$, we increase the exposure gradually from $0.04\,\mathrm{s}$ towards $0.5\,\mathrm{s}$ at $\Phi = 3 \times 10^{-3}$ ph $\mathrm{s}^{-1}\,\mathrm{nm}^{-2}$ to keep a sufficient SNR also at lower fluences.

In Figs. S2 and S3 the increase of the scattered intensity with increasing fluence and cooking temperature is shown. The intensity of the samples in the range 50 to $60\,^{\circ}\mathrm{C}$ is more than one order of magnitude lower compared to the samples at higher temperatures. For a sufficient SNR in a single measurement on these samples, a higher exposure time and increased number of frames would be necessary, but this does not allow to record the dynamics of the sample that are fast due to the absence of a stable gel network. This is the reason why the samples 50 to $60\,^{\circ}\mathrm{C}$ are not included in the XPCS analysis in the main text.

XPCS analyses

TTC Cutting

We extract $g^{(2)}(q,\tau)$ functions via horizontal cuts starting at the diagonal of the TTC at different starting times t_2 and define $\tau = t_1 - t_2$ with $t_1 \geq t_2$. For increasing the SNR, we average over several cuts along the t_2-axis. In the analysis of the long measurements with the highest attenuator (abs. 24, $\Phi = 0.003\,\mathrm{ph\,s^{-1}\,nm^{-2}}$,$\mathscr{D} = 0.002\,\mathrm{kGy\,s^{-1}}$), the time interval for averaging is 200 s, for the short measurements at high fluences, the time interval for averaging is one second. For all other settings, the time interval is 20 s noting that it could have been decreased to even smaller intervals towards higher fluences. Due to the slightly different starting times on the diagonal of the TTC, the $g^{(2)}(q,\tau)$ cuts have different lengths. This is handled by cutting the longer $g^{(2)}$s to the length of the cut with fewest data points such that a parallelogram-shaped region of the TTC is obtained and the issue of averaging with nan-type values is avoided.

$g^{(2)}$ Fitting

The correlation functions are modeled by a Kohlrausch-Williams-Watts (KWW) function [4]:

$$g^{(2)}(q,\tau) = 1 + \beta(q)\,e^{-2(\Gamma(q)\tau)^k}\,, \tag{2}$$

where $\beta(q)$ is the q-dependent speckle contrast [5], $\Gamma(q)$ is the decay rate and k is the KWW exponent containing information about the type of motion [6, 7]. The contrast is determined from measurements on a static aerogel sample ($\beta(q = 0.006\,\mathrm{nm^{-1}}) = 10.6\,\%$,$\beta(q = 0.02\,\mathrm{nm^{-1}}) = 9.7\,\%$). In some of our measurements on egg white, this contrast was not reached, which might be due to a second decay on short time scales that was not covered by the experiment. We handled this by setting the aerogel contrast as an upper boundary for the fits and fit the contrast as a third parameter in addition to $\Gamma(q)$ and $k(q)$. Especially at high q values (e.g. $q = 0.02\,\mathrm{nm^{-1}}$), the correlation functions do not decay to one, but to some finite offset b. This is due to the width of the q annulus, d$q = 0.004\,\mathrm{nm^{-1}}$, and the differences of the scattered intensity at the inner and outer edge of the q annulus. This is solved by adding an offset b to equation 2 that is fixed during the fitting procedure. If the correlation functions are not fully decaying in the measured time frame (Fig. S8), b is estimated from the aerogel contrast.

In some measurements (75 °C,$\mathscr{D} \geq 1\,\mathrm{kGy\,s^{-1}}$ and 78 °C,$\mathscr{D} \geq 2\,\mathrm{kGy\,s^{-1}}$), the radiation-induced dynamics display a double exponential decay. Those were fitted with a linear combi-

nation of two KWW functions:

$$g^{(2)}(q,\tau) = 1 + b + \beta \left(\beta_0 \, e^{-2(\Gamma_1 \tau)^{\alpha_1}} + (1-\beta_0) \, e^{-2(\Gamma_2 \tau)^{\alpha_2}}\right), \tag{3}$$

where β_0 gives the relative amplitude of each decay and the index 1 indicates the faster decay. An example for such a double-exponential decay is shown in Fig. S9. For the multiplication with the dose-dependent decay rate in Fig. S6 we use the slower decay that matches $k = 1.5$. The 1 kGy-cuts from these measurements could be fitted well with a single exponential.

Additional Information on Fig. 4

Figure 4 in the main text shows dose and dose rate effects on the relaxation rates Γ of egg white gel samples prepared at temperatures 63 °C(a), 70 °C(b), 73 °C(c) and 80 °C(d). Equivalents for other preparation temperatures can be found in Fig. S12. There we present the plot for the 73 °C-sample at $q = 0.006\,\mathrm{nm}^{-1}$ from which one can see that the differences in dose effects are not due to the increase of q for the analysis of the samples ≥ 73 °C.

The correlation functions that were fitted for this figure were obtained from horizontal cuts through the TTCs presented in Fig. S10 and S11.

For the highest fluences/dose rates in (a) and (b), $\Gamma(\mathcal{D})$ curves from two measurements are displayed: one with a short exposure time of 0.005 s to resolve effects of small doses and one with a higher exposure time of 0.04 s to catch effects at high doses. The two measurements were performed at different spots of the sample, but they do not align perfectly, and the first points of the long measurements are significantly lower than the last points of the short measurement (e.g. red curves in Fig. 4a). This might be due to some opening effects of the detector's fast shutter in the first frames of the measurement or because of radicals traveling through the sample volume which might increase the effective dose of these measurements. But without further knowledge on the radicals, these effects are hard to estimate.

Fluidization of Egg White Gel

In Fig. 4a,b in the main text, we observe an increase in Γ accompanied by a decrease of the KWW exponent to values ≤ 1 which we attributed to a fluidization of the gel. This is further supported when looking at the behavior of Γ as a function of q for higher starting doses. In Fig. S13 we compare the $\Gamma(q)$ plots of different starting doses (a:1 kGy, b:10 kGy) for an egg white sample prepared at 63 °C and measured with $\mathscr{D} = 0.09\,\mathrm{kGy\,s}^{-1}$. The low dose cut displays the $\Gamma \propto q$ behavior known for gels [8], while for the higher dose, a q^2-term needs to be introduced, pointing towards diffusive motion.

Additional Information on Fig. 5

Figure 5 in the main text shows TTCs from a continuous illumination of a single spot on an egg white sample prepared at $80\,^{\circ}\mathrm{C}$ with a change of dose rates during the exposure. The displayed TTCs are the end of a longer measurement where the amplitude of the dose rate switches was gradually increased. Therefore the sample has already accumulated a dose of $\approx 80\,\mathrm{kGy}$. The measurement was performed on the same capillary as those in Fig. S12, but $\approx 8\,\mathrm{h}$ after sample preparation. So aging effects cause differences in sample dynamics between the two figures.

Between the TTCs in Fig. 5 in the main text, there is a time gap of eleven seconds, which corresponds to the time needed for file storage and change of absorbers.

Additional Information on Fig. 6

Figure 6 in the main text shows a comparison of fluence (dose rate) effects on the dynamics of egg white gel samples at a constant starting dose of $1\,\mathrm{kGy}$. As explained before, all correlation functions are averages over several neighboring cuts of the TTC. For Fig. 6, the cuts were taken such that the average dose accumulated at the starting point of the cut is $1\,\mathrm{kGy}$. We used $1\,\mathrm{kGy}$ instead of a smaller dose to avoid possible effects of shutter opening especially in the measurements with high fluences were large doses are accumulated within seconds.

We separated the curves of the different samples in two distinct plots according to the different response of dynamics and structure to dose and the given labels *soft* gel network and *strong* gel network. For the strong gel networks, the measurements with absorber 24 are left out due to the effect seen in Fig. S10. In the absorber 18 measurements the precision of the fit results of the decay rates was not high enough for a reasonable velocity value.

Panels c and d of Fig. 6 show the accumulated dose values at which the $g^{(2)}$ functions have decayed to a value of $g^{(2)}(t') = 1 + \beta \exp(-2)$. The starting dose of the cut of $1\,\mathrm{kGy}$ is not added here to resolve the change in panel c, but has to be added when calculating dose threshold values.

Stress Relaxation Model

We model the stress relaxations in our protein gels as a series of N consecutive relaxation events as proposed by [9–12]. The intermediate scattering function can be written as a sum over relaxation events $f(q,t) = \sum_N P(N,t)h(q,N)$ where the Poisson distribution $P(N,t) = \exp(-\gamma t)(\gamma t)^N/N!$ gives the probability of N relaxation events occurring with a rate γ during a time interval t. The term $h(q,N)$ describes the degree of decorrelation that is introduced by N events. We assume ballistic motion, such that the directions of the N displacements are the

same and a Gaussian distribution of the displacements which gives $h(q, N) \approx \exp(-(qN\delta)^2)$ [12]. Here, δ is the average displacement of a single relaxation.

In Fig. S14 we model correlation functions via $g^{(2)}(q, \tau) = 1 + \beta|f(q, \tau)|^2$ for twelve values of $q\delta$ between 0.01 and 1. The sum is performed over $N = 200$ consecutive relaxation events occurring with a rate $\gamma = 120\,\mathrm{s}^{-1}$. The resulting correlation functions are fitted with a KWW function (equation 2). From the results for the KWW-exponent k in Fig. S14b, we infer values of $q\delta \approx$ 0.01 - 0.1. The fit results for the relaxation rate Γ in Fig. S14c show that in the same regime of $q\delta$, Γ can be approximated by $\gamma q\delta$, implying that the typical decorrelation rate Γ of the $g^{(2)}$ functions is 10 to 100 times smaller than the microscopic stress relaxation rate γ.

Details on Estimation of Radical Formation Rates

We estimate the density of radicals generated in the irradiated sample volume that are necessary for the instantaneous switching of dynamics under different dose rates observed in Fig. 5 in the main text. We assume that this instantaneous acceleration occurs already at the threshold fluence Φ_D determined in Fig. 6 in the main text and that the X-ray absorption of the egg white protein gel, that has a water content of $\approx 88\%$ [13], is comparable to the absorption in water ($A = 72\%$ in $1.5\,\mathrm{mm}$ of water). Thus, the absorption rate of photons is

$$R_{\mathrm{ph}} = \Phi_D \cdot A \cdot z^2, \tag{4}$$

where z^2 is the size of the X-ray beam, that is $z^2 = 100 \times 100\mathrm{\mu m}^2$ in our experiment. The energy of a single photon is E_{ph} =8.54keV which gives an energy absorption rate of

$$R_E = R_{\mathrm{ph}} \cdot E_{\mathrm{ph}}. \tag{5}$$

Typically, an absorption of $100\,\mathrm{eV}$ generates three radicals [14]. Using this, the rate of radical formation becomes

$$R_{\mathrm{radical}} = R_E \cdot \frac{3}{100\,\mathrm{eV}}. \tag{6}$$

In the last step we divide the radical formation rate by the irradiated sample volume $z^2 d$, where $d = 1.5\,\mathrm{mm}$ is the diameter of the capillary. Absorption in the glass or diffusion of radicals out of the irradiated volume are not taken into account. This leads to

$$r_{\mathrm{radical}} = \frac{R_{\mathrm{radical}}}{z^2 d}. \tag{7}$$

In Table S1 we present the results and intermediate steps for a typical soft egg white gel ($\Phi_D = 0.003\ \mathrm{ph\,s^{-1}\,nm^{-2}}$) and a strong egg white gel ($\Phi_D = 0.9\ \mathrm{ph\,s^{-1}\,nm^{-2}}$). We want to obtain

Table S1: Calculation of radical formation rates

	soft gel network	strong gel network
$\Phi_D(\mathrm{ph\,s^{-1}\,nm^{-2}})$	0.003	0.9
$R_{\mathrm{ph}}(\mathrm{ph\,s^{-1}})$	2.2×10^7	6.5×10^9
$R_E(\mathrm{eV\,s^{-1}})$	1.9×10^{11}	5.5×10^{13}
$R_{\mathrm{radical}}(\mathrm{radicals\,s^{-1}})$	5.7×10^9	1.6×10^{12}
$r_{\mathrm{radical}}(\mathrm{radicals\,s^{-1}\,nm^{-3}})$	3.8×10^{-7}	1.1×10^{-4}

a lower limit for the radical density. Therefore we calculate for simplicity the radical density after one second of illumination which is twice the exposure time of a single frame in Fig. 5 where we do not observe any region of transition from slow to fast dynamics in the TTC. Thus the radicals effect the dynamics already on timescales below $0.5\,\mathrm{s}$.

After one second of illumination with Φ_D, the soft egg white gel has accumulated $\approx 3.8 \times 10^{-7}$ radicals nm^{-3} which equals one radical per cube of $138\,\mathrm{nm}$. In the strong gel $\approx 1.1 \times 10^{-4}$ radicals nm^{-3} were generated, which is one radical per cube of $20\,\mathrm{nm}$.

α Values from other Works

A linear dependence of decay rate Γ on the applied fluence/dose rate has been observed in other glasses and gels as well. Therefore we can extract values for α and compare them to the ones found in egg white. The data on protein glasses are taken from Fig. 2a,c in the work of Chushkin et al. [15] where highly crowded solutions of α-crystalline were investigated in the region of a glass transition. For the translation of dose rate into fluence, we assumed water-like absorption and a beam size of $25 \times 25\mu\mathrm{m}^2$. For the translation of Γ into v, we used $q = 1\,\mathrm{nm}^{-1}$. The data for oxide glasses are taken from the Fig. 2d of Ruta et al. [16] using $q = 15\,\mathrm{nm}^{-1}$. The data on hydrated lysozyme stem from the Fig. 1c in the work of Bin et al. [17]. The data on IgG PEG were measured at 5 °C in the same experimental setup as the egg white data sets. Details and raw data can be obtained from the authors upon request. The IgG PEG system is known to approach a glass transition at low temperatures [18] which is why it is included in the category "proteins near glass transitions" in Fig. 8 in the main text.

Investigation of Aging Effects

Gel systems are known to display aging effects. We investigated possible effects of aging by illuminating three different sample spots for each fluence. The data are from a different beam time in the same setup and in contrast to the data set presented in the main text, the egg white was prepared with a 50mM NaCl. The resulting TTCs are displayed in Fig. S16 and show a good

reproducibility of the dynamics and an absence of significant aging effects in the time frame of the measurements. But we observe a fast slow-down in the first scan at the lowest absorber of the strong gel network that was also observed in other first measurements on strong gel networks and needs further investigation (Fig. S10).

Supporting References

1. Falus, P., Lurio, L. B. & Mochrie, S. G. J. Optimizing the Signal-to-Noise Ratio for X-ray Photon Correlation Spectroscopy. *Journal of Synchrotron Radiation* **13,** 253–259 (2006).
2. Möller, J., Sprung, M., Madsen, A. & Gutt, C. X-Ray Photon Correlation Spectroscopy of Protein Dynamics at Nearly Diffraction-Limited Storage Rings. *IUCrJ* **6,** 794–803 (2019).
3. Reiser, M. *et al.* Resolving Molecular Diffusion and Aggregation of Antibody Proteins with Megahertz X-ray Free-Electron Laser Pulses. *Nature Communications* **13,** 5528 (2022).
4. Williams, G. & Watts, D. C. Non-Symmetrical Dielectric Relaxation Behaviour Arising from a Simple Empirical Decay Function. *Transactions of the Faraday Society* **66,** 80 (1970).
5. Hruszkewycz, S. O. *et al.* High Contrast X-ray Speckle from Atomic-Scale Order in Liquids and Glasses. *Physical Review Letters* **109,** 185502 (2012).
6. Perakis, F. & Gutt, C. Towards Molecular Movies with X-ray Photon Correlation Spectroscopy. *Physical Chemistry Chemical Physics* **22,** 19443–19453 (2020).
7. Lehmkühler, F., Roseker, W. & Grübel, G. From Femtoseconds to Hours—Measuring Dynamics over 18 Orders of Magnitude with Coherent X-rays. *Applied Sciences* **11,** 6179 (2021).
8. Duri, A. & Cipelletti, L. Length Scale Dependence of Dynamical Heterogeneity in a Colloidal Fractal Gel. *Europhysics Letters (EPL)* **76,** 972–978 (2006).
9. Cipelletti, L., Manley, S., Ball, R. C. & Weitz, D. A. Universal Aging Features in the Restructuring of Fractal Colloidal Gels. *Physical Review Letters* **84,** 2275–2278 (2000).
10. Bouchaud, J.-P. & Pitard, E. Anomalous Dynamical Light Scattering in Soft Glassy Gels. *The European Physical Journal E* **6,** 231–236 (2001).
11. Duri, A., Bissig, H., Trappe, V. & Cipelletti, L. Time-Resolved-Correlation Measurements of Temporally Heterogeneous Dynamics. *Physical Review E* **72,** 051401 (2005).
12. Caronna, C., Chushkin, Y., Madsen, A. & Cupane, A. Dynamics of Nanoparticles in a Supercooled Liquid. *Physical Review Letters* **100,** 055702 (2008).
13. Mine, Y. Recent Advances in the Understanding of Egg White Protein Functionality. *Trends in Food Science & Technology* **6,** 225–232 (1995).
14. Schwarz, H. A. Free Radicals Generated by Radiolysis of Aqueous Solutions. *Journal of Chemical Education* **58,** 101 (1981).
15. Chushkin, Y. *et al.* Probing Cage Relaxation in Concentrated Protein Solutions by X-Ray Photon Correlation Spectroscopy. *Physical Review Letters* **129,** 238001 (2022).

16. Ruta, B. *et al.* Hard X-rays as Pump and Probe of Atomic Motion in Oxide Glasses. *Scientific Reports* **7,** 3962 (2017).

17. Bin, M. *et al.* Coherent X-ray Scattering Reveals Nanoscale Fluctuations in Hydrated Proteins. *The Journal of Physical Chemistry B* **127,** 4922–4930 (2023).

18. Girelli, A. *et al.* Microscopic Dynamics of Liquid-Liquid Phase Separation and Domain Coarsening in a Protein Solution Revealed by X-Ray Photon Correlation Spectroscopy. *Physical Review Letters* **126,** 138004 (2021).

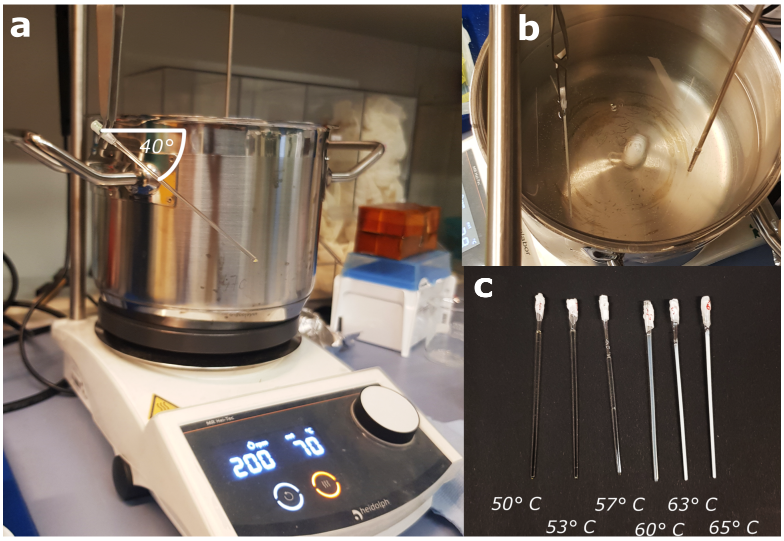

Figure S1: **Setup for cooking egg white samples. a** Side view in front of the cooking setup to show the angle of the capillary. **b** Top view with magnetic stirrer. **c** Cooked samples at temperatures 50 to 65 °C.

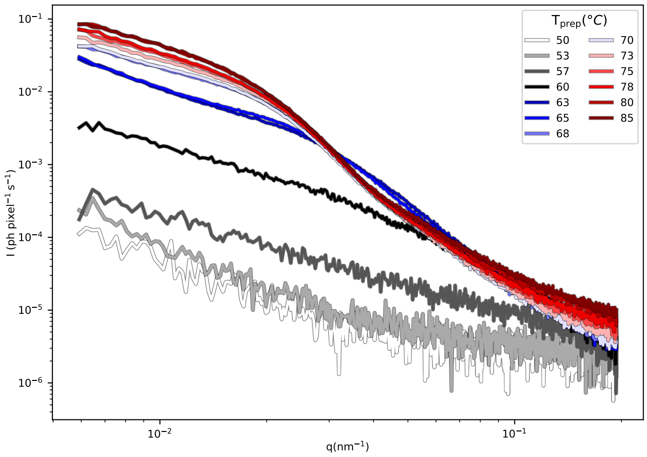

Figure S2: USAXS profiles of all measured samples recorded at a low dose rate of 10 Gy s^{-1}. The displayed profiles are averages over the first 100 frames (0.5 to 20 s depending on exposure time) taken in every measurement to reduce noise.

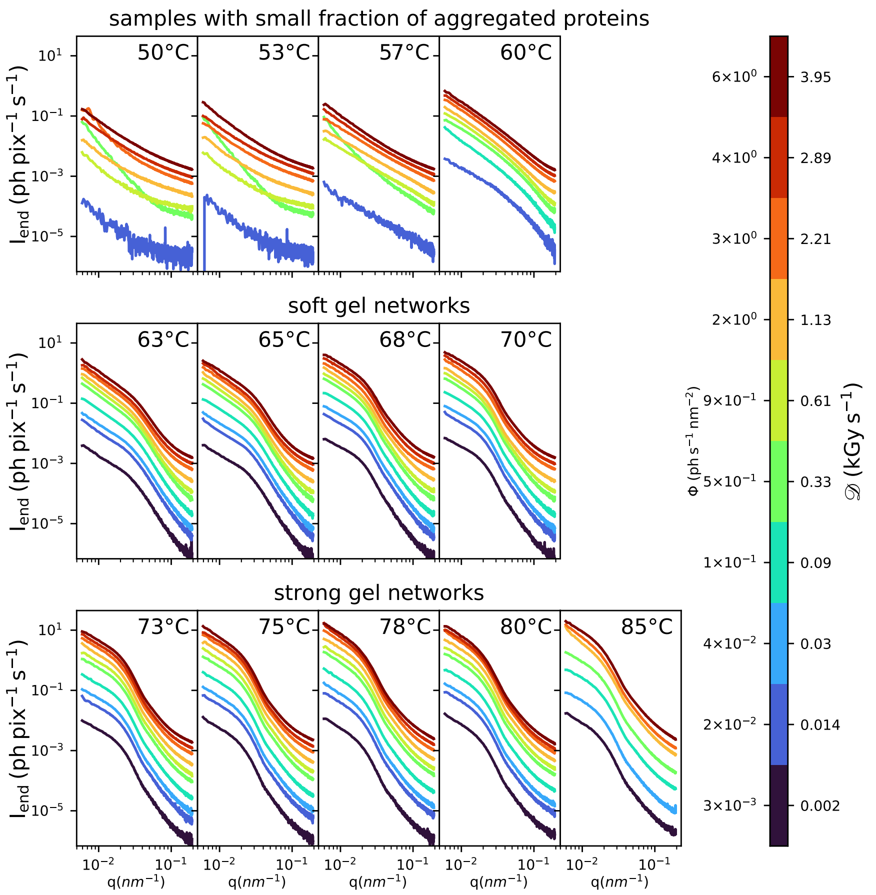

Figure S3: Comparison of scattered intensity at the end of every measurement to determine samples where an XPCS analysis is possible. The scattered intensity increases both with cooking temperature (direct comparison in Fig. S2) and with incoming flux.

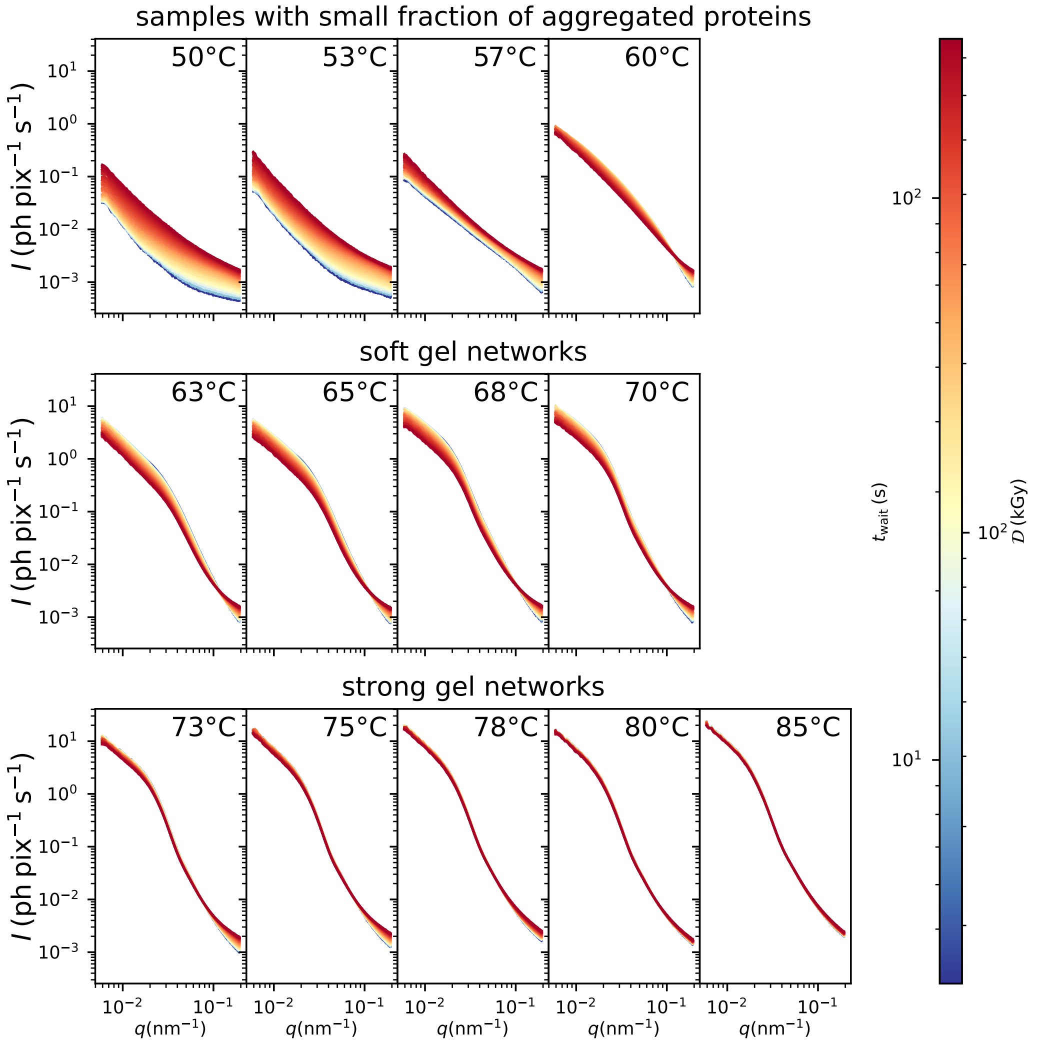

Figure S4: Evolution of the scattered intensity I as a function of momentum transfer q upon increasing dose measured with $\mathscr{D} = 4\,\mathrm{kGy\,s^{-1}}$,$\Phi = 6\,\mathrm{ph\,s^{-1}\,nm^{-2}}$.

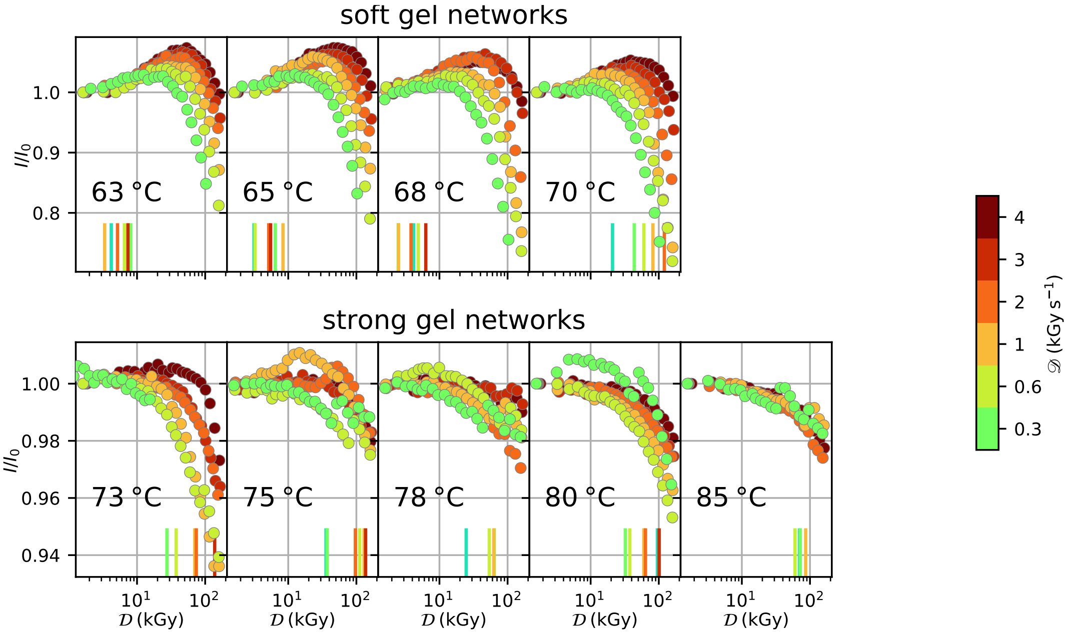

Figure S5: Relative increase of the scattered intensity in the q-range 0.006 to $0.03\,\mathrm{nm}^{-1}$ as a function of accumulated dose $\mathcal{D}$. The vertical lines mark the dose thresholds where the intensity exceeds a deviation of $\pm 1\,\%$.

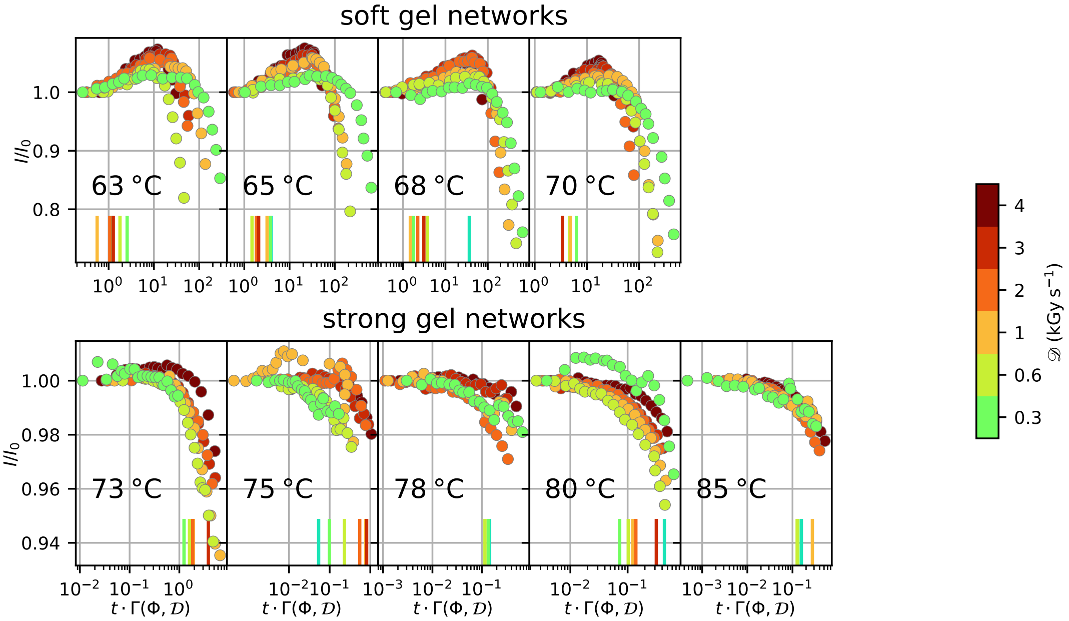

Figure S6: Relative increase of the scattered intensity in the q-range 0.006 to $0.03\,\mathrm{nm}^{-1}$ as a function of the product of the measurement time and dose dependent decay rate. The decay rates are taken from Fig. S12. The vertical lines mark the thresholds where the intensity exceeds a deviation of $\pm 1\,\%$.

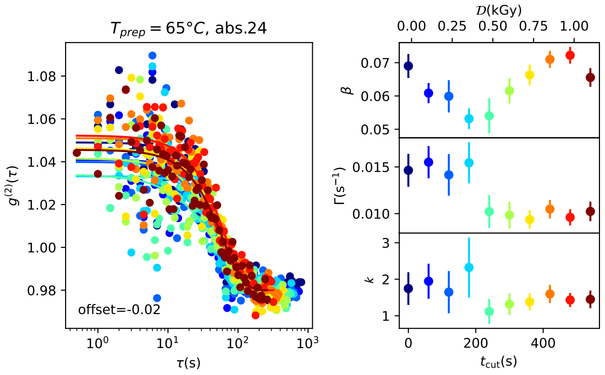

Figure S7: Example for KWW-fit for a sample prepared at 65° and measured with the lowest fluence (abs. 24, $\Phi = 0.003\mathrm{ph\,s^{-1}\,nm^{-2}}$). Due to the low number of scattered photons, the correlation function decays below 1. We decided to fit it anyway as the decay is clearly visible. The panels on the right show the results for the three free fit parameters: contrast β, decay rate Γ and KWW exponent k.

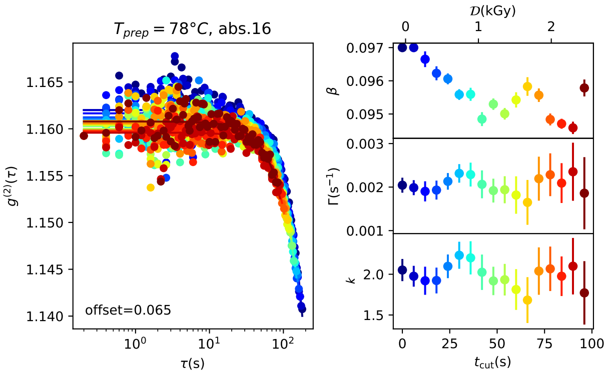

Figure S8: Example for KWW-fit a the sample prepared at 78° and measured with a fluence of $\Phi = 0.04\text{ph s}^{-1}\text{ nm}^{-2}$ (abs. 16). Due to the slow dynamics, the correlation function does not decay completely within the time frame of the measurement. We know the offset of 0.065 from measurements with higher fluence where the decay shifts into the experimental time frame. The panels on the right show the results for the three free fit parameters: contrast β, decay rate Γ and KWW exponent k.

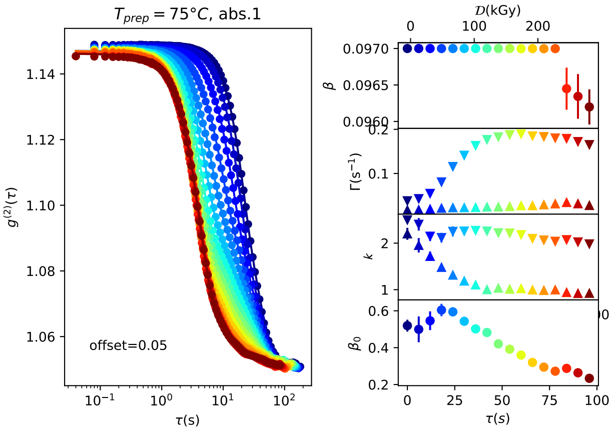

Figure S9: Example for KWW-fit a the sample prepared at 75° and measured with high fluence (abs.1, $\Phi = 4.4\text{ ph s}^{-1}\text{ nm}^{-2}$). Upon increasing dose, the correlation functions displays a second decay. The panels on the right show the results for the six free fit parameters: contrast β, two decay rates Γ, the corresponding KWW exponents k and the relative amplitude of the slower decay β_0. The fit results for the slower decay are indicated with upper triangles, the results for the faster decay with lower triangles. It can be seen that the aerogel contrast of 9.7% was set as an upper limit for the contrast.

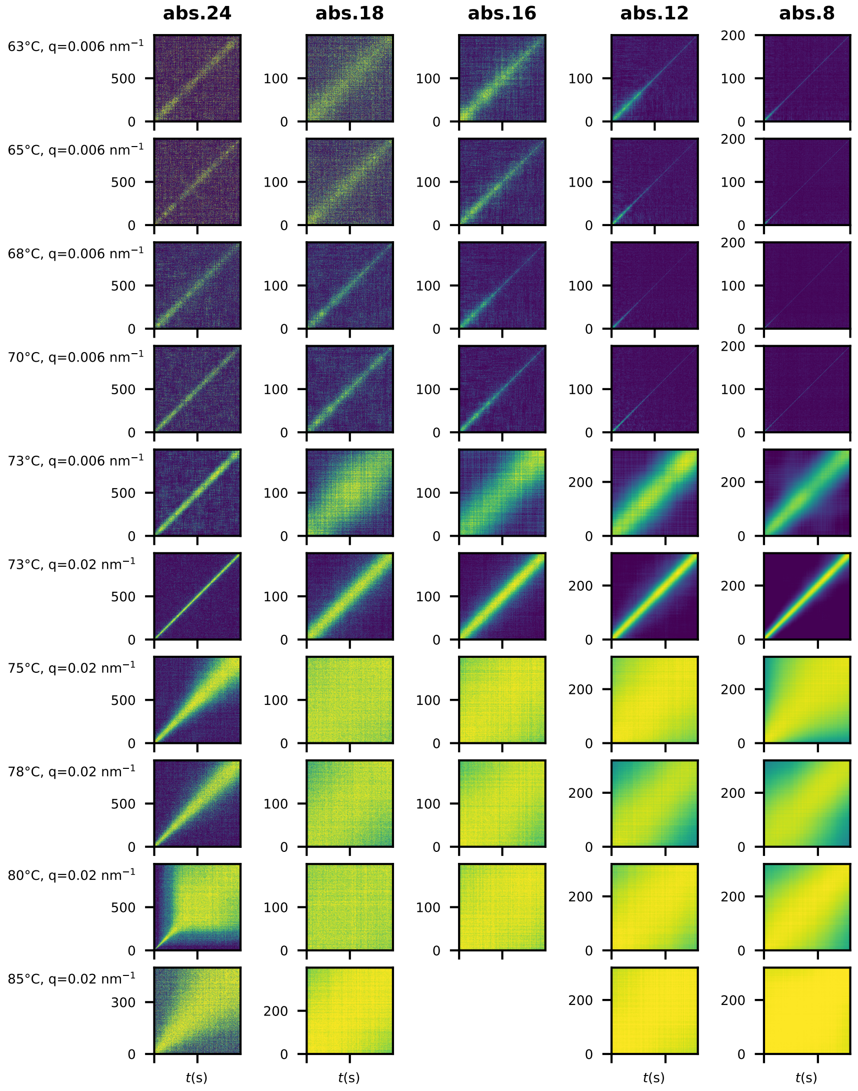

Figure S10: Overview of TTCs recorded at the lower fluences for all temperatures. The range of the colorbar was set such that it spans the maximum range of contrast determined with the aerogel sample. For the sample prepared at 73 °C, the results for both q values are displayed. The first recorded TTCs at absorber 24 are significantly faster and the origin is not yet clear. Therefore these measurements are left out in the analysis. The sample prepared at 85 °C was not measured with absorber 16.

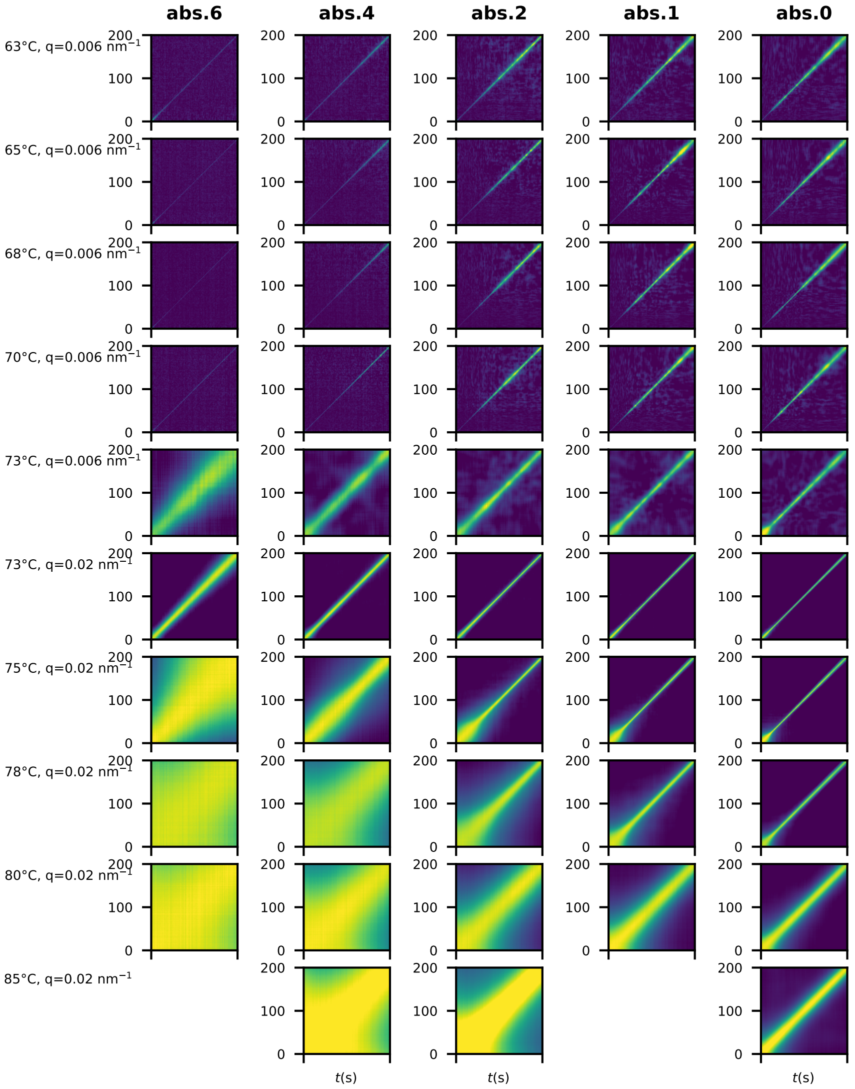

Figure S11: Overview of TTCs recorded at the higher fluences for all temperatures. The range of the colorbar was set such that it spans the maximum range of contrast determined with the aerogel sample. For the sample prepared at 73 °C, the results for both q values are displayed. Only the long measurements with 0.4 s exposure time are shown here for clarity. The sample prepared at 85 °C was not measured with absorbers 6 and 2.

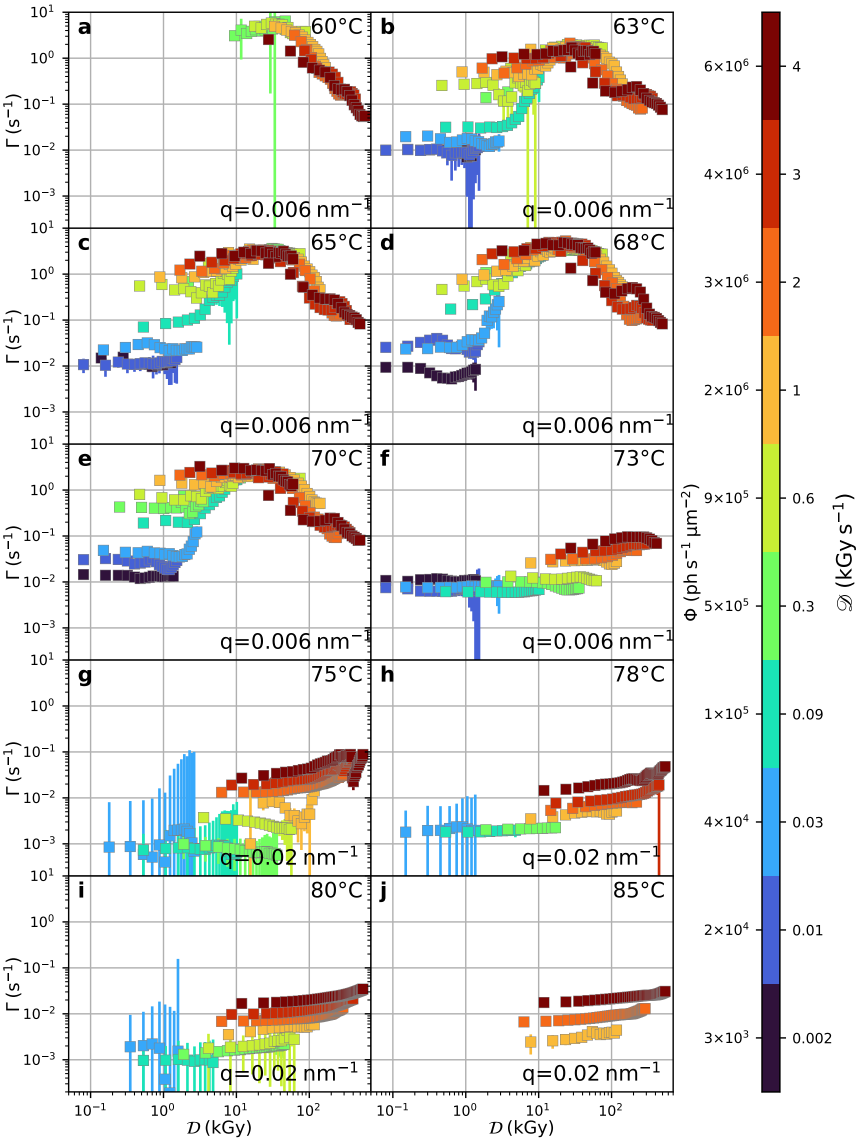

Figure S12: Decay rates Γ as a function of dose and dose rate for samples where an XPCS analysis was feasible. At 60 °C the SNR was not sufficient at low photon fluences. In contrast to Fig. 4 in the main text, the $\Gamma(\mathcal{D})$-curve for the sample prepared at 85 °C is shown for $q = 0.006\,\mathrm{nm}^{-1}$.

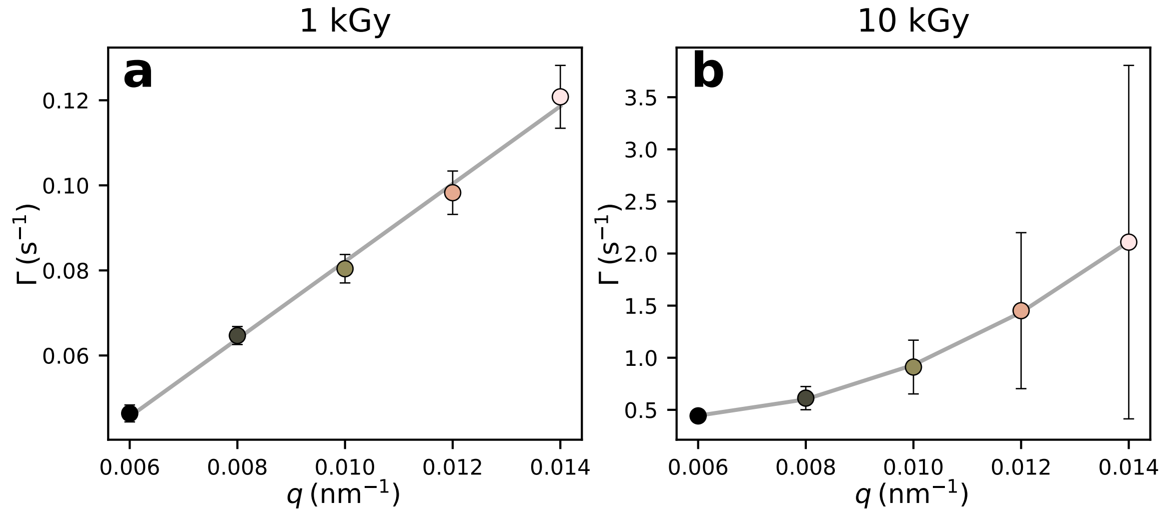

Figure S13: Dependence of the decay rate Γ on the wave vector transfer q found at $g^{(2)}$ cuts starting at 1 kGy (a) and 10 kGy (b) in an egg white sample prepared at 63 °C and measured with a dose rate of $\mathscr{D} = 0.09 \mathrm{kGy\,s^{-1}}$. In (a) the gray line represents a linear fit. In (b) the points are fitted with a second order polynomial.

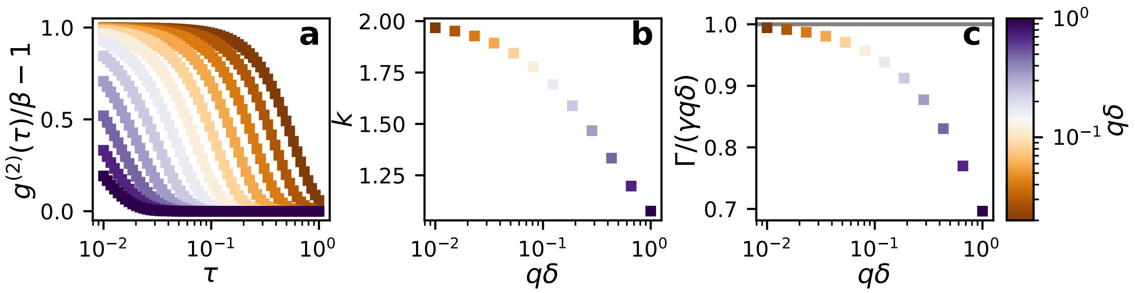

Figure S14: Calculated $g^{(2)}$ functions according to the model discussed in the text representing a superposition of $N = 200$ relaxation events with a rate $\gamma = 120$. **a** $g^{(2)}$ functions for twelve values of $q\delta$ indicated by the different colors. Solid lines are fits with KWW functions. **b** Fit results for KWW exponents k. **c** Fit results for relaxation rate Γ normalized to the rate of relaxation events γ and $q\delta$.

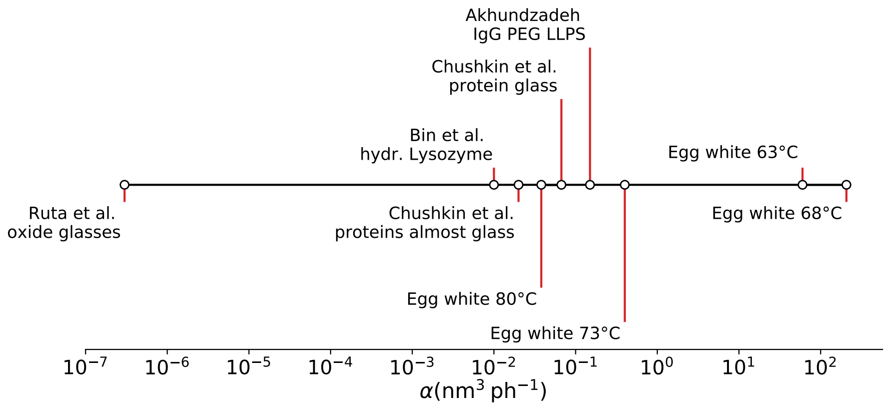

Figure S15: Comparison of α parameters describing the strength of the X-ray matter interaction that causes the acceleration of the data. Data are estimated from the work of Chushkin et al. [15], the work of Bin et al. [17], and the work of Ruta et al. [16].

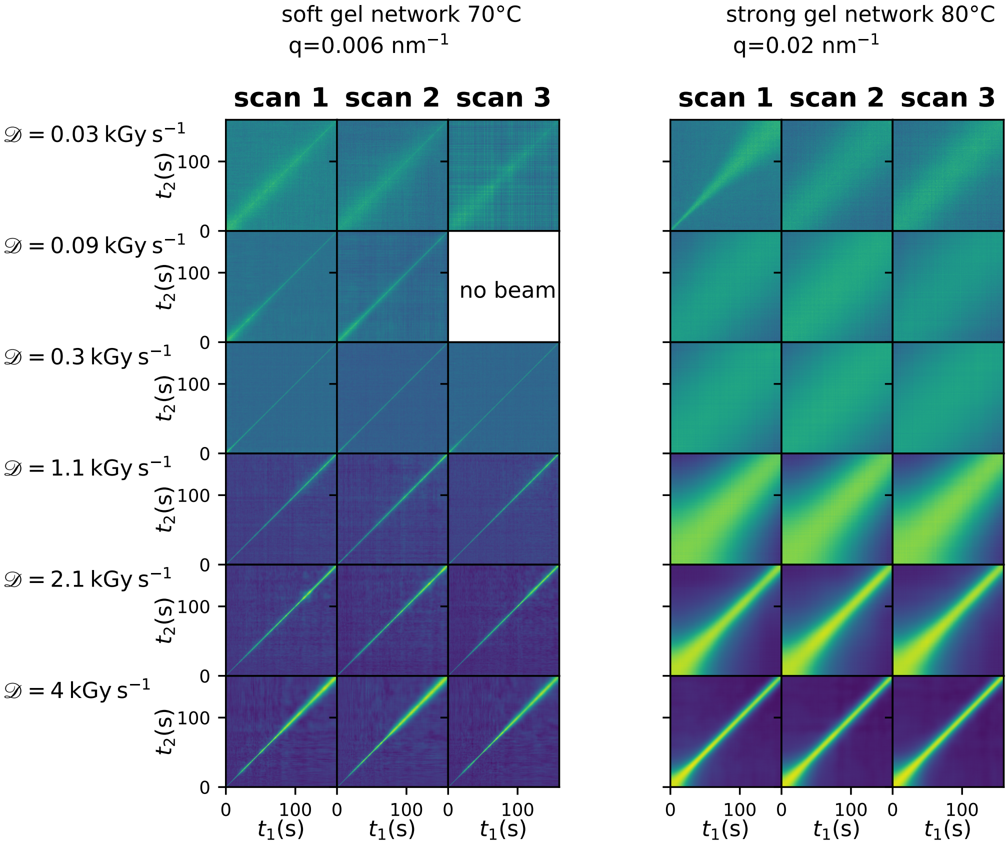

Figure S16: Test of aging effects for two different egg white gels by taking three consecutive measurements on different spots on the sample with six different dose rates/fluences.
